# Well-Defined Amylose Acetate-*graft*-polylactide Graft Polymers as Compatibilizers for Renewable Polymer Blends

**DOI:** 10.1021/acs.biomac.5c01188

**Published:** 2025-09-26

**Authors:** Jeffrey E. Thompson, Isabela T. Coutinho, Nicholas F. Pietra, Louis A. Madsen, Robert B. Moore, Kevin J. Edgar

**Affiliations:** 1 Macromolecules Innovation Institute, Virginia Tech, Blacksburg, Virginia 24061, United States; 2 Department of Chemistry, Virginia Tech, Blacksburg, Virginia 24061, United States; 3 Department of Sustainable Biomaterials, Virginia Tech, Blacksburg, Virginia 24061, United States

## Abstract

Regioselectively substituted amylose acetate-*graft*-polylactide (AmAc-*g*-PLA) graft polymers were synthesized via grafting-to “click” reaction between C6-azide functionalized AmAc and alkyne-terminated PLA. Alkyne-terminated PLA synthesized through organocatalytic ring-opening polymerization (ROP) permitted control over graft degree of polymerization (DP) and stereochemistry, while azide functionalized AmAc with tailorable C6-azide degree of substitution (DS) allowed graft density control. This describes the first synthesis of polysaccharide-based graft polymers with exclusive C6 grafting and controllable topology. Thermal analysis indicates that glass transition temperatures (*T*_g_) of AmAc and PLA segments are affected after grafting-to coupling, with poly­(l-lactide) (PLLA) grafts maintaining crystallizability. AmAc-*graft*-poly­(d,l-lactide) (PDLLA) graft polymers were effective compatibilizers for immiscible blends of starch acetate (StAc) and PDLLA as evidenced by small-angle laser light scattering (SALLS) and phase contrast optical microscopy (PCOM). This method permits the determination of structure–property relationships with regard to the effect of graft polymer topology on blend compatibilization, which will be invaluable in designing compatibilized biobased polymer blends as sustainable materials.

## Graphical abstract

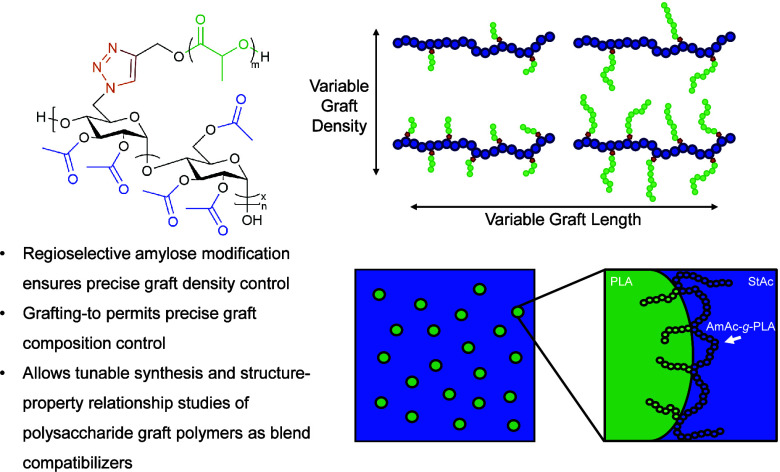

## 1 Introduction

Concerns over introduction of petroleum-based plastic waste, leading to microplastics in the environment, fuel the need to replace such nondegradable materials. Recent bans on nondegradable plastics for grocery bags and other single-use applications drive significant interest in developing renewably sourced and/or environmentally degradable (i.e., compostable) materials to substitute for synthetic plastics. Such sustainable materials include compostable polyesters sourced from petroleum feedstocks (i.e., poly­(ε-caprolactone) (PCL), poly­(butylene succinate) (PBS)), compostable polyesters sourced from renewable feedstocks (i.e., poly­(lactic acid) (PLA), poly-3-hydroxybutyrate), or naturally occurring biopolymers (i.e., proteins, polysaccharides) and their derivatives (i.e., starch esters, cellulose esters).^1^ However, the sustainable materials mentioned above tend to have higher production costs, lower toughness, in some cases inadequate melt processability, and higher water sensitivity versus traditional petroleum-sourced plastics, hindering their widespread use in such applications.^2^ A common approach to improve these shortcomings is through physical blending with another polymer with complementary properties. However, most polymer pairs are thermodynamically immiscible, causing poor physical properties arising from the lack of adhesion between phase-separated domains in the blended system.^3^

Properties of immiscible polymer blends can at times be improved by adding a well-designed graft polymer compatibilizer. These comprise two polymers, each miscible with one of the blend components, allowing the compatibilizer to sit at the interface of immiscible phases, reduce interfacial tension, enhance phase adhesion, and better stabilize the phase-separated morphology to improve physicochemical properties.^4^ Optimal blend compatibilization can synergistically enhance properties including impact resistance^5^ and tensile strength,^6^ generating blended systems with superior properties compared to their constituent components. Possibly the most ubiquitous compatibilized polymer blend is high-impact polystyrene (PS), which contains a glassy PS matrix with dispersed rubbery polybutadiene (PBD) phases containing a PBD-*g*-PS graft polymer compatibilizer.^7^

Some graft polymers where the polysaccharide is the backbone and a different polymer comprises the grafted chains have been studied as immiscible blend compatibilizers. The plethora of hydroxy groups on each monosaccharide are conducive to producing graft polymer structures, as these can be initiating sites for ring-opening polymerization^8^ (ROP) or can be substituted with initiating species for controlled radical polymerizations.^9,10^ The biodegradable nature of polysaccharides and the drive to generate compostable materials spur significant research on the synthesis of aliphatic polyester-grafted polysaccharides (many aliphatic polyesters are also biodegradable)^11^ as blend compatibilizers. Examples of such compatibilizers include cellulose triacetate (CTA)-*g*-poly­(l-lactide) (PLLA) and CTA-*g*-poly­(D,l-lactide) (PDLLA) for CTA/PLLA blends,^12^ cellulose acetate (CA)-*g*-PDLLA for CA/PLLA blends,^13^ amylose-*g*-PDLLA for starch/PLA blends,^14^ starch-*g*-PCL for starch/PCL blends,^15^ and dextran-*g*-PCL for starch/PCL blends.^16^ Other polysaccharide-based blend compatibilization strategies include addition of amphiphilic plasticizers,^17^ cross-linking agents,^18^ and the incorporation of a third, distinct polymeric component, such as the addition of poly­(vinyl acetate-*co*-vinyl alcohol) to thermoplastic starch (where crystalline starch granules are disrupted by thermal treatment with a plasticizer, permitting thermal processability) (TPS)/PLLA blends^19^ or the addition of a PBS-based copolymer to wheat flour/PBS blends.^20^ While effective at compatibilizing immiscible polysaccharide-based blends, these methods do not exert significant microstructural or morphological control of resulting blended systems. To understand structure–property relationships between graft polymer topology and polysaccharide-based blend morphology, regioselective chemistries must be employed to generate well-defined polysaccharide graft polymers with tunable microstructures.

Synthesis of polysaccharide graft polymers with regioselective substitution of polymeric side chains is difficult due to the presence of multiple hydroxy groups possessing low and comparable reactivities.^21^ Despite this, various regioselective and chemoselective methods have been employed to generate well-defined structures. The Tsujii group prepared regioselectively substituted 2,3-di-*O*-methyl-6-PS cellulose via regioselective tritylation of the C6 position, followed by permethylation, deprotection, and *N*-(3-(dimethylamino)­propyl)-*N’*-ethylcarbodiimide hydrochloride (EDC·HCl) coupling of the deprotected C6-OH with 4-pentynoic acid. Subsequent grafting-to employing copper-catalyzed azide–alkyne cycloaddition (CuAAC) with an azide-terminated PS generated a regioselectively substituted cellulosic bottlebrush polymer, which was examined by small-angle X-ray scattering (SAXS) and size-exclusion chromatography with multiangle light scattering (SEC-MALS) to study its solution conformation.^22^ A similar approach generated 2,3-di-*O*-PCL-6-PS^23^ and 2,3-di-*O*-poly­(ethylene glycol) (PEG)-6-PS^24^ cellulosic bottlebrush polymers, which were to study solution dynamics and microphase separation of Janus bottlebrushes, respectively.

Regioselectively substituted polysaccharide graft polymers can also be prepared by grafting-from methods, as demonstrated by Ifuku and Kadla, in which the authors prepared a 2,3-di-*O*-methyl-6-*O-*bromoisobutyryl cellulose macroinitiator for atom transfer radical polymerization (ATRP) with *N*-isopropylacrylamide (NIPAAm), generating a thermoresponsive 2,3-di-*O*-methyl-6-PNIPAAm cellulose graft polymer.^25^ In addition to cellulose, chitosan with polymer side chains grafted regioselectively at the C6 position^26^ and regioselectively (and chemoselectively) at the C2 primary amine have been prepared through both grafting-from^27^ and grafting-to^28^ approaches. While these synthetic methods produced polysaccharide graft (co)­polymers with high degrees of structural control, time-consuming protection and deprotection steps were required to ensure regioselectivity.

We recently reported synthesis of 2,3-di-*O*-acetyl-6-azido-6-deoxy (2,3Ac-6N_3_) amylose with exclusive azide incorporation at the C6 position, at controlled DS­(N_3_), as precursors to well-defined graft polymers, without employing protecting group strategies.^29^ We used a small molecule, *tert*-butyl propargyl ether (TBPE), as a proof-of-concept to demonstrate the ability to generate branched polymers with high control over branch density and location employing the CuAAC “click” reaction. Such methodology could be expanded to CuAAC grafting-to using an alkyne-terminated polymer, creating a pathway to regioselectively grafted polysaccharide graft polymers with a degree of microstructural control unusual in polysaccharide chemistry. Previous researchers observed that both graft length and graft density of graft polymer compatibilizers significantly affect both the morphology and thermomechanical properties of compatibilized blends.^6^ In particular, we believe that such well-defined graft polymers would allow for a systematic study of the effect of topological features on the ability of graft polymers to compatibilize immiscible blends. A deeper understanding of such structure–property relationships is vital to improve the range of physicochemical properties available to compatibilized polysaccharide-based blends, leading to more useful materials sourced from renewable feedstocks.

Herein, we describe regioselective synthesis and characterization of well-defined amylose acetate (AmAc)-*g*-PLA graft polymers via grafting-to CuAAC with PLA chains exclusively incorporated at the C6 position, by an approach that requires no protecting groups (Scheme 1). We hypothesized that this approach would achieve near-perfect regioselective incorporation of PLA branches, and approach complete control over vital parameters like graft length and density. By varying DS­(N_3_), DP­(PLA), and stereochemistry of lactide repeat units (l- vs D/L-), we sought to generate well-defined graft polymers where graft density, length, and stereochemistry could all be carefully tuned. Previous methods to prepare polysaccharide-based graft polymers have not approached the level of efficiency, control over graft density and length, and regio- and chemospecificity that we anticipated by this approach. We expected that these studies would guide us to effective graft polymer compatibilizers for immiscible blends of high DS­(Ac) starch acetate (StAc) and PLA, with graft polymer topological control allowing structure–property relationship studies. We envisioned that a compatibilized blend of StAc/PLA could be optimized to generate a less-expensive PLA-based material, possibly increasing its appeal as a more viable replacement to nondegradable plastics for single-use applications such as packaging or biodegradable cutlery.

**Scheme 1:**
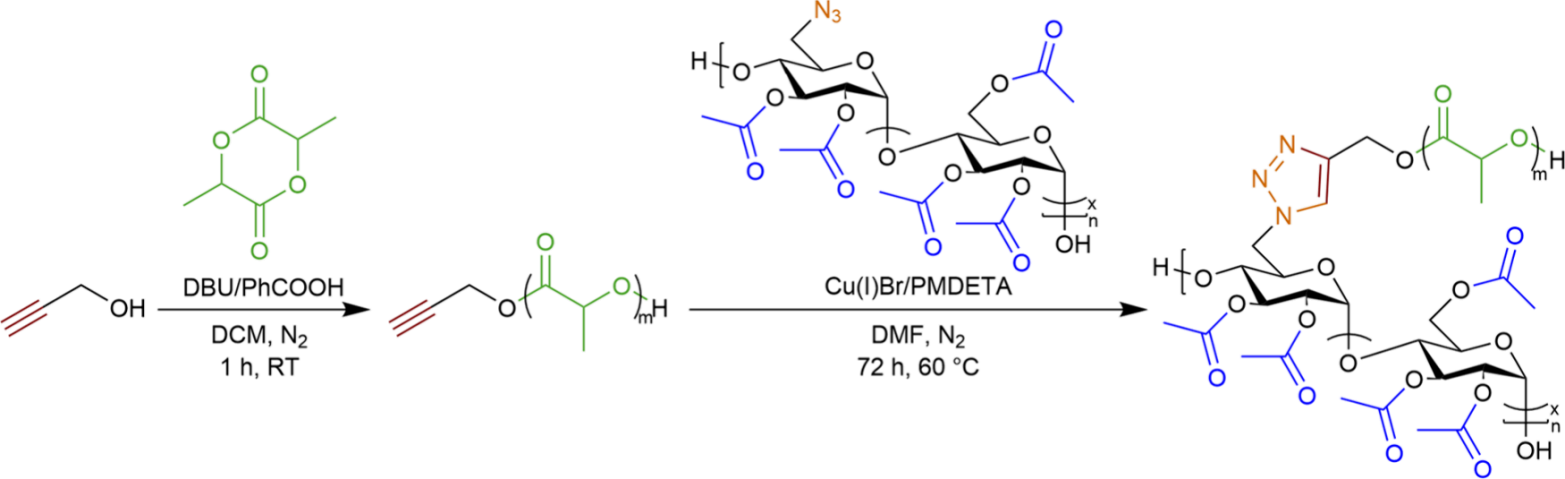
Synthetic Route to Regioselectively Substituted AmAc-*g*-PLA Graft Polymers with Tunable Graft Lengths and Densities

## 2 Experimental Section

### 2.1 Materials

Amylose isolated from potato starch (Biosynth, YA10257), with *M*_n_ = 1610 kg/mol (degree of polymerization (DP_n_) = 9940) and *Đ* = 4.12 (determined by size exclusion chromatography (SEC) of its tricarbanilate derivative),^30^ and starch (isolated from potato, Sigma) were dried at 50 °C under reduced pressure overnight before use. *N,N*-Dimethylacetamide (DMAc, Fisher), dimethyl sulfoxide (DMSO, Sigma), pyridine (Sigma), and tin­(II) 2-ethylhexanoate (SnOct_2_, Sigma) were stored over 4Å molecular sieves. Dichloromethane (DCM, Fisher) was dried over CaH_2_, distilled onto 3Å molecular sieves, and stored under dry N_2_ until use. CHCl_3_ (Fisher) was dried over P_2_O_5_, distilled onto 3Å molecular sieves, and stored under dry N_2_ until use. *N,N*-Dimethylformamide (DMF, Spectrum) and benzyl alcohol (BnOH, Sigma) were dried over CaH_2_, distilled under reduced pressure onto 4Å molecular sieves, and stored under dry N_2_ until use. Propargyl alcohol (Sigma), *N,N,N’,N’’,N’’*-pentamethyldiethylenetriamine (PMDETA, Oakwood Chemical), and 1,8-diazabicyclo[5.4.0]­undec-7-ene (DBU, Sigma) were dried over CaH_2_, distilled under reduced pressure onto 3Å molecular sieves, and stored under dry N_2_ until use. LiBr (Alfa Aesar) and NaN_3_ (Fisher) were dried overnight at 125 °C under reduced pressure and stored in a desiccator under vacuum until use. Benzoic acid (PhCOOH, Merck) was dried overnight at 80 °C under reduced pressure and stored in a desiccator under vacuum until use. Triphenylphosphine (PPh_3_, Sigma) was recrystallized from ethanol (EtOH) and dried at room temperature (RT) under reduced pressure for 2 d. *N*-Bromosuccinimide (NBS, Acros) was recrystallized from boiling water and dried at RT over anhydrous CaCl_2_ under reduced pressure for 2 d. L- and D,l-Lactide (ChemImpex) were recrystallized from ethyl acetate (EtOAc) and dried at RT under reduced pressure for 2 d. Cu­(I)Br (Sigma) was stored under dry N_2_ until use. Acetic anhydride (Ac_2_O, Acros), trifluoroacetic anhydride (TFAA, Sigma), acetic acid (AcOH, Fisher), phenyl isocyanate (Acros), NaI (Fisher), Merrifield resin (α-chloromethylstyrene supported on cross-linked polystyrene, 2.0–3.0 mmol -Cl/g, TCI), and Chelex 100 sodium form (sodium iminodiacetate supported on cross-linked polystyrene, 50–100 mesh, Sigma) were used as received. All other solvents and materials were of reagent grade and used as received.

### 2.2 Measurements

^1^H and ^13^C NMR spectra were obtained on a Bruker Avance II 500 MHz spectrometer equipped with a BBO Prodigy probe at RT using deuterated chloroform (CDCl_3_) as solvent. ^1^H NMR spectra for alkyne-terminated PLA were obtained on an Agilent MR4DD2 400 MHz spectrometer with a broadband OneNMR probe. ^1^H NMR spectra were obtained with at least 64 scans with 1 s delay and ^13^C NMR spectra were obtained using at least 4096 scans with 3 s delay. Diffusion-ordered spectroscopy (DOSY) experiments were performed using a Bruker Avance III 400 MHz/9.4 T wide-bore spectrometer equipped with a high gradient diffusion probe (Bruker Diff50) paired with a 5 mm ^1^H RF coil inset. The measurement used a 90° time of 4.5 μs, an acquisition time of 1 s, a repetition time of 2 s, δ = 1–1.5 ms, Δ = 30–50 ms, and a maximum gradient strength ranging from 300–900 G/cm. The values of these diffusion encoding parameters were selected to achieve ≥ 85% attenuation in 16 steps. Fourier transform infrared (FTIR) spectra were acquired using a Varian 670 IR spectrometer equipped with a Pike Technologies GladiATR attachment and collected as the average of 32 scans. Thermogravimetric analysis (TGA) was performed using a TA Instruments TGA 5500 at a heating rate of 20 °C/min up to 500 °C. Glass transition temperatures (*T*_g_) were obtained by differential scanning calorimetry (DSC) performed on a TA Instruments DSC Q2500. Approximately 5 mg samples in aluminum pans were heated at 10 °C/min up to approximately 30–50 °C below onset of decomposition, then cooled at 20 °C/min to 0 °C, then heated again at 10 °C/min. *T*_g_ values were obtained from the second heating cycle to erase any previous thermal history and were obtained in triplicate. The *T*_g_ was reported as the midpoint of the endothermic step transition, while the *T*_m_ was reported as the maximum of the melting endotherm. SEC-MALS characterization of PLA was carried out in THF (stabilized with 1 mM BHT) at 1 mL/min at 30 °C using a Shimadzu LC-20AD HPLC pump equipped with an Agilent PLgel MIXED 10 μm guard column, two Agilent PLgel 10 μm MIXED-B LS separation columns, a Wyatt DAWN HELEOS-II MALS detector, and a Wyatt Optilab T-rEX dRI detector. Samples were weighed into a glass vial, dissolved in the mobile phase, then filtered through a 0.22 μm PTFE syringe filter. Absolute molecular weights and dispersities were calculated with Wyatt ASTRA software and off-line d*n*/d*c* analysis, using a known PLA d*n*/d*c* value of 0.042 mL/g in THF. SEC-MALS characterization of StAc was performed in DMAc with 50 mM LiCl at 50 °C at a flow rate of 0.5 mL/min (Agilent isocratic pump, degasser, and autosampler, columns: TOSOH TSKgel Guard Alpha and TOSOH TSKgel Alpha-3000: molecular weight range 0–1 × 10^5^ g/mol). Detection consisted of a Wyatt Optilab refractive index (RI) detector operating at 785 nm, a Wyatt DAWN multiangle light scattering detector operating at 783 nm, and an Agilent MWD operating at 365 nm. Absolute molecular weights and dispersities were calculated using Wyatt ASTRA software and off-line d*n*/d*c* analysis, assuming 100% mass recovery. Films of 70/30 StAc/PDLLA blends with thickness below 10 μm, suitable for phase contrast optical microscopy (PCOM) and small-angle laser light scattering (SALLS), were prepared through solution casting. The polymer and compatibilizers were dissolved in CHCl_3_ (1 wt/v%) at RT until clear solutions were obtained. Then, 0.03 mL of the solution was cast onto microscope slides and evaporation over 15 min was controlled by covering the cast solution with a 25 mL Erlenmeyer flask. PCOM was used to characterize blend morphology. For this analysis, a Nikon Eclipse LV100 microscope in phase contrast mode equipped with an AmScope digital camera (MU503B) was used. The images were collected using a 20x Ph1 objective. Small-angle laser light scattering (SALLS) was used to measure blend interdomain distances. Scattering patterns were obtained in transmission with parallel polarizers (Vv mode) using a 3 mW He–Ne (λ = 632.8 nm) laser font and a AmScope digital camera (MU503B). The scattering vector (q) range was calibrated using a 300 grooves/mm diffraction grating with a sample-to-detector distance of 24 cm. The radially integrated scattering intensity as a function of the scattering vector (q) was obtained using SAXSGUI software and the interdomain distance (d) at the maximum intensity was calculated. Calculations for determining the diffusion coefficient (*D*), interdomain distance (d), Fox–Flory parameters, and PLA degree of substitution (DS­(PLA), i.e., graft density) are provided in the Supporting Information.

#### 2.3 Representative Synthesis of 2,3-Di-*O*-Acetyl-6-Azido-6-Deoxy (2,3Ac-6N_3_) Amylose

Synthesis and characterization of 2,3Ac-6N_3_ amylose was recently described.^29^ Briefly, dry amylose (2.50 g, 15.4 mmol anhydroglucose units (AGU)) was slurried in 100 mL anhydrous DMAc at 160 °C for 30 min under N_2_, after which 10.0 g dry LiBr was added to the flask followed by an additional 20 mL anhydrous DMAc. The mixture was stirred for an additional 10 min, after which approximately 20 mL DMAc was distilled under slight vacuum. The flask was backfilled with dry N_2_ and allowed to cool to RT, resulting in a transparent, amber solution. All solutions were kept under dry N_2_ until use within 24 h. Ac_2_O (14.6 mL, 154 mmol, 10 eq/AGU) was added quickly to the flask under dry N_2_ and allowed to mix at RT for 1 h. PPh_3_ (4.04 g, 15.4 mmol, 1 eq/AGU) and NBS (2.74 g, 15.4 mmol, 1 eq/AGU) were dissolved in separate 50 mL portions of dry DMAc. The PPh_3_ solution was added to the amylose solution over 5 min via syringe transfer, followed by the NBS solution, also over 5 min via syringe transfer, all under dry N_2_. The flask was lowered into an oil bath set at 70 °C and allowed to stir at that temperature for 1 h. The temperature was then raised to 80 °C, and the solution was stirred for an additional 32 h. The cooled solution was added to 2.5 L chilled 1:1 H_2_O:MeOH to precipitate the product, which was filtered and collected. The product was twice dissolved in acetone/EtOAc and twice reprecipitated in EtOH, being collected by filtration each time. The resulting product was dried overnight at 50 °C under reduced pressure to yield 2,3-di-*O*-acetyl-6-bromo-6-deoxy (2,3Ac-6Br) amylose (DS­(Ac) 2.88, DS­(Br) 0.12). Dry 2,3Ac-6Br amylose (DS­(Ac) 2.88, DS­(Br) 0.12, 2.50 g, 8.64 mmol AGU) was dissolved in 100 mL dry DMSO overnight at 40 °C. NaN_3_ (1.40 g, 2.5 eq/AGU) was added to the flask, and the solution was heated to 80 °C and stirred at that temperature for 24 h. The product was then poured into 1.5 L chilled MeOH and isolated by filtration. The product was redissolved in acetone/EtOAc and reprecipitated into EtOH, filtered, collected, and dried overnight at 50 °C under reduced pressure to yield 2,3Ac-6N_3_ amylose (DS­(Ac) 2.88, DS­(N_3_) 0.12) as a white powder. 2,3Ac-6N_3_ amyloses will be denoted as 2,3Ac-6N_3_ amylose_x/y_, where x = DS­(Ac) and y = DS­(N_3_). Yield: 2.05 g (82.6%). ^1^H NMR (500 MHz, CDCl_3_): 1.94–2.01 (C2/C3 -O–COCH_3_), 2.17 (C6 -O–COCH_3_), 3.50–3.62 (C6 −CH_2_–N_3_), 3.85–5.37 (H1–H5), 4.22–4.53 (C6 −CH_2_–O-COCH_3_). ^13^C NMR (125 MHz, CDCl_3_): 20.6–21.0 (C2/C3/C6 -O–COCH_3_), 51.2 (C6 −CH_2_–N_3_), 62.2 (C6 −CH_2_–OCOCH_3_), 69.0–72.9 (C2–C5), 95.7 (C1), 169.6–170.8 (C2/C3/C6 -O–COCH_3_).

#### 2.4 Representative Synthesis of Alkyne-Terminated PLA

To a flame-dried, N_2_-flushed 50 mL Schlenk flask equipped with a stir bar, D,l-lactide (2.50 g, 17.3 mmol, 100 equiv) and activated 3Å molecular sieves were added. The flask was then evacuated at 0.07 mbar for 2 h to remove trace water present, then backfilled with dry N_2_. Anhydrous DCM (24.5 mL) and propargyl alcohol (10.0 μL, 0.173 mmol, 1 equiv) were added to the flask under N_2_ and allowed to stir for 2 h to dry the solution. In a separate vial, DCM (1 mL), active 3Å molecular sieves, DBU (22.3 μL, 0.147 mmol, 0.85 equiv) and PhCOOH (6.4 mg, 0.026 mmol, 0.15 equiv) were added and allowed to sit for 2 h to allow the sieves to remove trace water present. Then, 500 μL of the catalyst solution was quickly added to the lactide solution under N_2_ to initiate polymerization at RT. After 1 h, the flask was exposed to air and the reaction was quenched with a few drops of AcOH. The polymer solution was added dropwise to 750 mL cold MeOH to precipitate the product. The final product was isolated by filtration and further washed with MeOH to yield a white solid. Entry PDLLA-15.4k, Table 4.1. Yield: 2.14 g (85.6%). ^1^H NMR (400 MHz, CDCl_3_): 1.54 (terminal −CH_3_), 1.58 (repeat unit −CH_3_), 2.49 (−O–CH_2_–C≡C–H), 4.35 (terminal −CH−), 4.72 (−O–CH_2_–C≡C–H), 5.15 (repeat unit −CH−).

#### 2.5 Representative Synthesis of AmAc-*g*-PLA

In a 25 mL Schlenk flask, 2,3Ac-6N_3_ amylose (DS­(Ac) 2.88, DS­(N_3_) 0.12, 40 mg, 0.139 mmol AGU, 0.017 mmol -N_3_) and PDLLA-15.4k (387 mg, 0.025 mmol, 1.5 mol eq per mol AmAc C6–N_3_) were dissolved in 10.425 mL anhydrous DMF. After dissolution, the polymer solution was sparged with dry N_2_ for 30 min. In a 1-dram vial equipped with a rubber septum, Cu­(I)Br (14.4 mg, 0.100 mmol, 6 eq per mol AmAc C6–N_3_) and PMDETA (41.9 μL, 0.201 mmol, 2.0 eq per mol Cu­(I)­Br, 6 eq per mol -N_3_) were dissolved in 1 mL anhydrous DMF and subsequently sparged with dry N_2_ for 10 min. After both solutions were degassed, 250 μL of the catalyst/ligand solution was added to the polymer solution under N_2_ through a N_2_-purged needle. The flask was then lowered into an oil bath set at 60 °C and allowed to stir at that temperature for 72 h. PS-N_3_ resin (500 mg, ∼ 1.00 mmol -N_3_, ∼ 40 eq per mol alkyne-PDLLA) was quickly added to the flask under dry N_2_, and the flask was subsequently sparged again with dry N_2_ for 10 min. The flask was then allowed to stir at 60 °C for another 72 h to allow unreacted alkyne-PDLLA to conjugate to the PS resin. Finally, the solution was exposed to air and filtered into a scintillation vial to remove the scavenging resin. Chelex resin was added to the vial and allowed to stir at RT for 1 d, with additional Chelex resin added until the solution turned colorless. The solution was diluted with EtOAc/DMF, filtered into a flask, concentrated via rotary evaporation, then added to EtOH to precipitate the product. The product was filtered, collected, redissolved in minimal CHCl_3_, then added to 1:1 EtOH:hexanes to precipitate the product. The product was filtered and further washed with EtOH and hexanes, collected, and dried overnight at 50 °C under reduced pressure to yield a white powder. AmAc-*g*-PLA graft polymers are denoted with the convention of AmAc_*x*_-*g*_*y*_-PLA-z, where x is DS­(Ac), y is DS­(PLA) (i.e., grafting density), and z is the *M*_n_ of PLA in kg/mol obtained by SEC in THF. Yield: 137 mg (45.9%). ^1^H NMR (500 MHz, CDCl_3_) 1.54–1.58 (PLA −CH_3_), 1.94–2.01 (AmAc C2/C3 -O–COCH_3_), 2.20 (AmAc C6 -O–COCH_3_), 3.54–5.38 (AmAc backbone), 4.22–4.53 (AmAc C6 −CH_2_–O-COCH_3_), 7.79 (triazole -N–CH = C−). ^13^C NMR (125 MHz, CDCl_3_) 16.7 (PLA −CH_3_), 16.8 (terminal PLA −CH_3_), 20.7–21.1 (AmAc C2/C3/C6 -O–COCH_3_), 50.1 (AmAc C6 −CH_2_–N-), 58.6 (PLA -C–CH_2_–O-(COCHCH_3_O)_n_-), 62.4 (AmAc C6 −CH_2_–OCOCH_3_), 66.8 (terminal PLA −CH–OH), 69.0–73.0 (AmAc C2–C5 −CH−), 69.1 (PLA −CH−), 95.7 (AmAc C1 −CH−), 126.3 (triazole -N–CH = C−), 141.9 (triazole -N–CH = C−), 169.5–169.8 (PLA -O–COCH−), 169.6–170.8 (C2/C3/C6 -O–COCH_3_).

#### 2.6 Bulk ROP of d,l-Lactide

To a flame-dried, N_2_-flushed 100 mL 2-neck round-bottom flask equipped with a magnetic stir bar and glass stopcock, D,l-lactide (20.0 g, 139 mmol, 700 equiv) was added. The flask was evacuated under vacuum (0.07 mbar, 30 min) and backfilled with dry N_2_ three times. To the flask was added a 5.00 M solution of BnOH in toluene (0.198 mmol, 1 equiv) and a 0.752 M solution of SnOct_2_ in toluene (0.139 mmol, 0.7 eq, 0.1 mol % of monomer) under dry N_2_. The flask was lowered into an oil bath set at 160 °C and allowed to stir for 40 min, after which the temperature was increased to 180 °C and the melt was allowed to stir for an additional 20 min. The flask was then removed from heat, the contents diluted with CHCl_3_, and the solution was poured into chilled MeOH to precipitate the product. The product was isolated by filtration and dried overnight at 50 °C under reduced pressure to yield an off-white solid. Yield: 19.21 g (96.1%). ^1^H NMR (400 MHz, CDCl_3_) 1.58 (repeat unit −CH_3_), 4.49 (benzyl −CH_2_−) 5.15 (repeat unit −CH−), 7.16–7.37 (benzyl −CH).

#### 2.7 Synthesis of Starch Acetate (StAc)

In a 3-neck round-bottom flask equipped with a mechanical stirrer, reflux condenser, and liquid addition funnel, dry starch (10.0 g, 61.7 mmol AGU) was slurried in AcOH (31.7 mL, 555 mmol, 9.00 eq/AGU) at 110 °C for 2 h. The slurry was allowed to cool to RT, after which TFAA (55.8 mL, 401 mmol, 6.50 eq/AGU) was added dropwise over 1 h via a liquid addition funnel. After all TFAA was added, the solution was heated to 70 °C, where the light, opaque slurry slowly turned into a viscous, dark brown solution. After 1.5 h, the solution was cooled to RT, diluted with acetone, and added to 1.8 L cold MeOH to precipitate the product. The product was filtered, washed with H_2_O and additional MeOH, and collected. The product was suspended in acetone, added to 1:1 H_2_O:MeOH, filtered, collected, and dried overnight at reduced pressure at 50 °C to yield a white solid. Yield: 17.02 g (96.6%). ^1^H NMR (500 MHz, CDCl_3_) 1.95–1.99 (C2/C3 -O–COCH_3_), 2.19 (C6 -O–COCH_3_), 3.93–5.37 (H1–H6/6’). ^13^C NMR (125 MHz, CDCl_3_) 20.7–21.0 (C2/C3/C6 -O–COCH_3_), 62.3 (C6), 69.0–73.0 (C2–C5), 95.7 (C1), 169.6–170.9 (C2/C3/C6 -O–COCH_3_).

## 3 Results and Discussion

### 3.1 Preparation of 2,3Ac-6N_3_ Amylose with Varying DS­(N_3_): Tuning Graft Density

We recently reported regioselective synthesis of 2,3Ac-6N_3_ amylose with DS­(N_3_) 0.04–0.99 as a functionalized backbone suitable for CuAAC modification.^29^ The high degree of structural control over both azide functionalization density and functionalization position (incorporated only at the C6 position, ensuring stoichiometric control up to a maximum of only 1 azide moiety per AGU), position 2,3Ac-6N_3_ amylose as an ideal candidate for preparing polysaccharide graft polymers with tunable graft densities. Synthesis and characterization of 2,3Ac-6N_3_ amylose as well as proof-of-concept of its CuAAC reactivity with a representative small molecule, TBPE, were described in that study and will not be discussed here in depth. Of particular interest for graft polymer compatibilizers are 2,3Ac-6N_3_ amylose with DS­(N_3_) 0.04, 0.12, and 0.19. Assuming full conversion of grafting-to CuAAC, the resulting graft polymers would have maximum grafting densities of 4%, 12%, and 19%, corresponding to an AmAc backbone with PLA grafts occurring approximately every 25, 8, and 5 repeating units, respectively. We hypothesize that compatibilization efficacy of AmAc-*g*-PLA will improve with decreasing graft density, permitting for more favorable enthalpic interactions between the StAc phase (predominantly consisting of triacetylated AGU repeating units) and ungrafted AmAc repeat units (also comprising triacetylated AGU repeating units). As such, we focused this work on preparing a library of AmAc-*g*-PLA graft polymers with grafting densities <20%. Graft densities lower than 4% can be achieved through treating excess 2,3Ac-6N_3_ amylose DS­(Ac) 2.96/DS­(N_3_) 0.04 with alkyne-terminated PLA, meaning that some azide residues will remain unmodified, which we do not believe will interfere with compatibilization results.

### 3.2 Preparation of Alkyne-Terminated PLA: Tuning Graft Length

We sought to prepare a series of PLAs end-functionalized with an alkynyl moiety as CuAAC partners for grafting-to graft polymer synthesis with 2,3Ac-6N_3_ amylose. Employing grafting-to chemistry allows stringent control over both DP_n_ and molar mass dispersity (*Đ*) of grafted side chains, as well as ready characterization thereof, allowing for greater compositional control vs grafting-from ROP. The grafting-to method also ensures that PLA grafts will only be appended at the C6 position of amylose, guaranteeing a maximum of one PLA graft at each monosaccharide repeat unit. It should be noted that, even though we observed nearly quantitative substitution of amylose hydroxy groups with either an acetyl or azido moiety,^29^ there still might be some free hydroxy groups present, which could act as undesired initiating sites for grafting-from ROP of lactide, further emphasizing the value of the C6 grafting-to CuAAC approach to previous endeavors.

We employed propargyl alcohol as initiator under anhydrous conditions to ensure each PLA chain contained a terminal alkynyl functionality suitable for CuAAC. PLA can be synthesized using various organic or organometallic catalysts, with the predominant method being bulk ROP catalyzed by tin­(II) 2-ethylhexanoate (SnOct_2_). Although previous studies indicated that lactide ROP catalyzed by SnOct_2_ can generate PLA with high molecular weight (MW) and low *Đ*,^31^ possible intramolecular backbiting and transesterification could potentially result in products lacking alkyne termini, preventing CuAAC reactivity.^32^ Synthesis of PLA using SnOct_2_ as catalyst is also typically conducted at high temperatures (∼120–180 °C), raising concerns on whether we would get full initiation with propargyl alcohol due to its relatively lower boiling point (*T*_b_ = 114 °C). We instead used a 1,8-diazabicyclo[5.4.0]­undec-7-ene (DBU) and benzoic acid (PhCOOH) catalyst system due to its high activity, fast kinetics at RT (permitting solution polymerization in DCM), low *Đ,* and low transesterification tendency.^33^

We prepared a series of alkyne-terminated PLAs with varying MW and tacticity (both racemic PDLLA and stereopure PLLA) through lactide ROP initiated by propargyl alcohol using DBU/PhCOOH as a highly active organocatalytic pair. Successful synthesis of alkyne-terminated PLA was confirmed via ^1^H NMR spectroscopy (Figure 1A). Resonances associated with the PLA repeating unit methyl (D, 1.58 ppm), repeating unit methine (C, 5.15 ppm), terminal methyl (F, 1.54 ppm), and terminal methine (E, 4.35 ppm) were all readily identifiable and agreed with previous literature.^34^ The terminal alkynyl functionality was confirmed by the characteristic methine triplet (A) at 2.49 ppm, indicating successful incorporation of the CuAAC reactive handle. Integral value ratios of alkyne methine (I­(A)) and terminal methine of the last lactyl repeat unit (I­(E)) ranged from 0.93:1.00 to 0.99:1.00, indicating that nearly all PLA chains were initiated by propargyl alcohol, rather than adventitious water that may have been present during polymerization (Table 1). It should be noted that prevention of PLA initiation by water during ROP is difficult, even under anhydrous conditions.^12^

**Figure 1:**
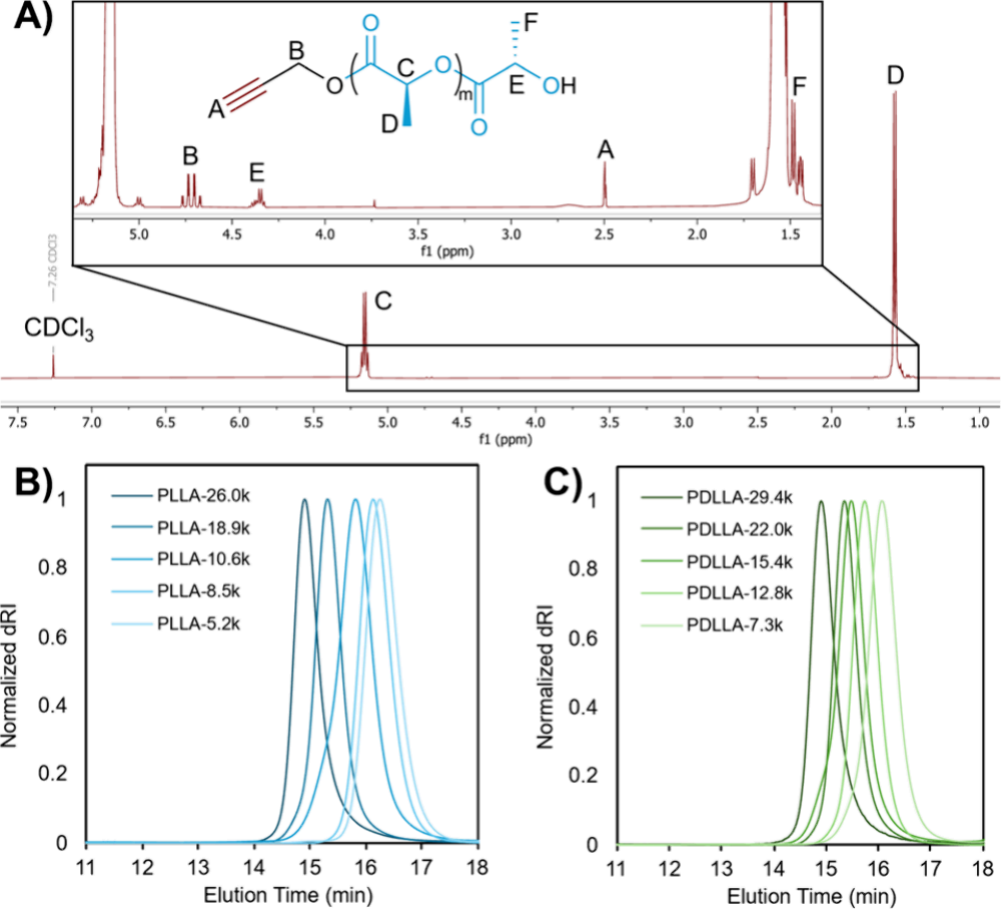
A) ^1^H NMR spectrum of PLLA-8.5k, B) SEC chromatograms of alkyne-PLLA, and C) SEC chromatograms of alkyne-PDLLA.

**Table 1:** Results of ROP of l- and d-,l-Lactide with Propargyl Alcohol and DBU/PhCOOH

sample	*M*_n_ (^1^H NMR, kg/mol)	*M*_n_ (SEC, kg/mol)	*Đ*	I(A)/I(E)	*T*_g_ (°C)
PLLA-5.2k	4.8	5.2	1.03	0.98	48.1
PLLA-8.5k	8.8	8.5	1.09	0.93	50.5
PLLA-10.6k	9.4	10.6	1.09	0.96	51.9
PLLA-18.9k	18.5	18.9	1.03	0.99	53.7
PLLA-26.0k	23.9	26.0	1.02	0.98	55.8
PDLLA-7.3k	7.9	7.3	1.07	0.96	49.6
PDLLA-12.8k	12.3	12.8	1.08	0.93	51.4
PDLLA-15.4k	16.5	15.4	1.07	0.95	52.6
PDLLA-22.0k	18.8	22.0	1.02	0.98	52.9
PDLLA-29.4k	27.7	29.4	1.01	0.93	54.5

Carefully tuning graft lengths of graft polymers necessitates control over DP and *Đ* of alkyne-terminated PLA. DP control allows for good control over the PLA graft length, while maintaining low *Đ* ensures that each arm is approximately the same length, permitting precise evaluation of structure–property relationships. Since PLA has a molecular weight for entanglement (*M*_e_) of approximately 8.7 kg/mol,^35^ we chose to prepare alkyne-terminated PLAs with number-average molecular weights (*M*_n_) below, near, and above its *M*_e_, believing that increasing the graft length of PLA side chains past *M*_e_ would allow for entanglements between pendant side chains of AmAc-*g*-PLA and the PLA phase in StAc/PLA blends. We prepared a series of alkyne-terminated PLAs (both L- and D/L-) with *M*_n_ ranging from 5.2–29.4 kg/mol, all with narrow MW distributions (*Đ* ≤ 1.09) (Figures 1B and 1C, Table 1). Additionally, there was good agreement between *M*_n_ obtained from SEC and from ^1^H NMR end-group analysis, providing further evidence that this approach affords good chain-end fidelity and negligible competing polymerization initiation from trace water.

### 3.3 CuAAC of PLA and 2,3Ac-6N_3_ Amylose: Tunable Synthesis of AmAc-*g*-PLA

With functionalized AmAc backbone and alkyne-terminated PLA in hand, we then performed grafting-to CuAAC to synthesize AmAc-*g*-PLA with varying graft density, graft length, and graft stereochemistry. We performed these syntheses under anaerobic conditions in anhydrous DMF using excess alkyne-terminated PLA per 6-N_3_ moiety using a Cu­(I)­Br/PMDETA catalyst/ligand system to ensure full conversion of the AmAc azido moiety to the desired triazole. Our first attempts to synthesize AmAc-*g*-PLA resulted in physical gelation, potentially due to the high concentration of final graft polymer in solution (∼150 mg/mL). Subsequent grafting-to reactions were conducted at a total polymer concentration of 40 mg/mL and no gelation was observed.

While formation of graft polymers via grafting-to allows for high degree of control over the attached side chain, separation of the product from unreacted side chains (when used in excess relative to the polymer backbone) can be challenging. Typically, PLA homopolymer can be separated from crude products of starch-*g*-PLA graft polymers via extraction with toluene^36,37^ or acetone;^38,39^ however, our synthesized graft polymers exhibited solubility in both solvents (likely due to increased hydrophobicity due to polyester grafts and acetyl substitution of hydroxy groups), rendering this approach ineffective. Dialysis is effective at separating unreacted side chains from the desired graft polymer,^40^ but this method is both solvent and time intensive, and requires a significant difference in MW between the graft polymer and ungrafted side chain. Selective precipitation (in which the product mixture is added to a solvent which dissolves unreacted PLA but precipitates AmAc-*g*-PLA) was also not feasible due to the similar solubilities of PLA, 2,3Ac-6N_3_ amylose, and AmAc-*g*-PLA. To circumvent the issues associated with the previous methods, we utilized an azide-functionalized scavenging resin. After reacting alkyne-PLA and 2,3Ac-6N_3_ under CuAAC conditions for 3 d, PS-N_3_ resin was added to the flask under N_2_ flow, which was subsequently degassed again and run under CuAAC conditions for an additional 3 d. The excess, ungrafted PLA would react with the PS-N_3_ resin, which could then easily be separated from the desired graft polymer in solution by simple filtration. The appearance of carbonyl absorption bands in the FTIR spectra of scavenging resin indicated successful conjugation of alkyne-PLA to the PS beads (Figure S1). Residual Cu­(I) catalyst was removed by treating the AmAc-*g*-PLA solution with Chelex resin for 1 d under atmospheric conditions until the solution became transparent and colorless. Subsequent dilution with EtOAc/DMF, filtration, rotary evaporation, and precipitation into EtOH generated a white, solid product.

Successful grafting-to CuAAC was confirmed by ^1^H NMR and FTIR spectroscopy. Figure 2 shows the stacked ^1^H NMR spectra of 2,3Ac-6N_3_ amylose_2.96/0.04_, PLLA-18.9k, and AmAc_2.96_-*g*_0.04_-PLLA-18.9k. The terminal alkyne PLA methine resonance at 2.49 ppm disappeared after CuAAC, along with the formation of a new triazole methine resonance at 7.79 ppm, supporting successful azide–alkyne cycloaddition. Characteristic resonances associated with the AmAc AGU backbone (3.92–5.27 ppm), AmAc acetyl methyl groups (1.95–2.18 ppm), PLA repeating unit methyl groups (1.58 ppm), and PLA repeating unit methine groups (5.15 ppm) all were apparent in the product spectrum. The presence of these resonances, as well as the disappearance of the terminal alkyne methine and appearance of triazole methine resonances indicate that alkyne-PLA was successfully conjugated to 2,3Ac-6N_3_ amylose via grafting-to CuAAC and was consistent for other graft polymer samples (Figures S2 and S3). DS calculations also indicated that grafting-to CuAAC went to full conversion when an excess of alkyne-terminated PLA was used, while lower DS­(PLA) could be targeted by having azide moieties present in excess relative to the alkyne. This permitted generation of AmAc_2.96_-*g*-PDLLA graft polymers with DS­(PLA) 0.01 and 0.005, which were expected to be effective blend compatibilizers due to the lower graft density.^13^C NMR further supported successful CuAAC linking alkyne-terminated PLA and azide-functionalized 2,3Ac-6N_3_ amylose, with resonances associated with AmAc C6–N- methylene (50.1 ppm), PLA initiator methylene (58.6 ppm), triazole methine (126.3 ppm), and triazole quaternary carbon (141.9 ppm) all readily identifiable (Figure S4).

**Figure 2:**
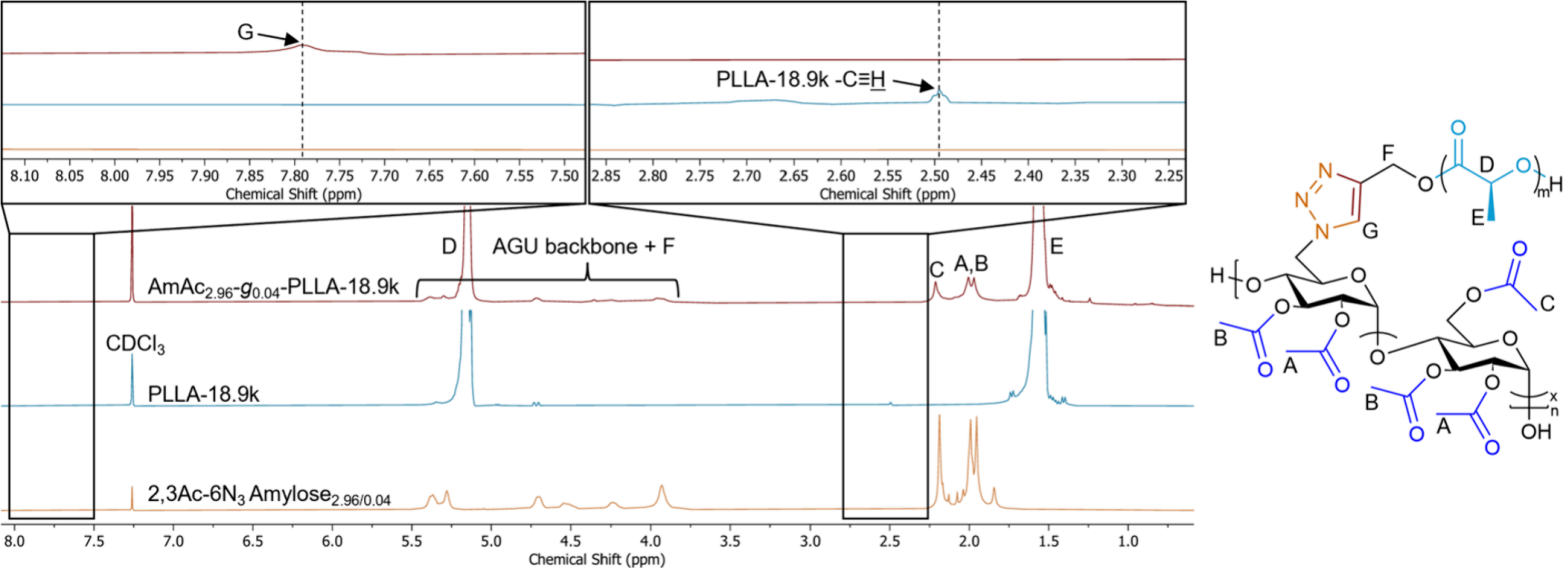
Stacked ^1^H NMR spectra of 2,3Ac-6N_3_ amylose_2.96/0.04_, PLLA-18.9k, and AmAc_2.96_-*g*_0.04_-PLLA-18.9k.

FTIR spectroscopy also supported successful grafting-to CuAAC, as the characteristic azide absorption band at 2103 cm^–1^ present in 2,3Ac-6N_3_ amylose was absent in the FTIR spectrum of AmAc-*g*-PLA, supporting complete cycloaddition to the corresponding triazole (Figures 3 and S5). Absorption bands corresponding to PLA methine stretching (2998 cm^–1^), AmAc C6-*O*-acetyl methylene asymmetric stretching (1231 cm^–1^), PLA −CH–CO-O- asymmetric stretching (1184 cm^–1^), PLA methyl asymmetric rocking (1131 cm^–1^), PLA −O–CH-CO- asymmetric stretching (1088 cm^–1^), AmAc acetal symmetric stretching (950 and 896 cm^–1^) and AmAc C6-*O*-acetyl methylene wagging (602 cm^–1^) modes were all identified in the FTIR spectrum of AmAc_2.81_-*g*_0.19_-PLLA-5.2k and agreed with previously identified FTIR absorptions for PLLA^41^ and StAc.^42^

**Figure 3:**
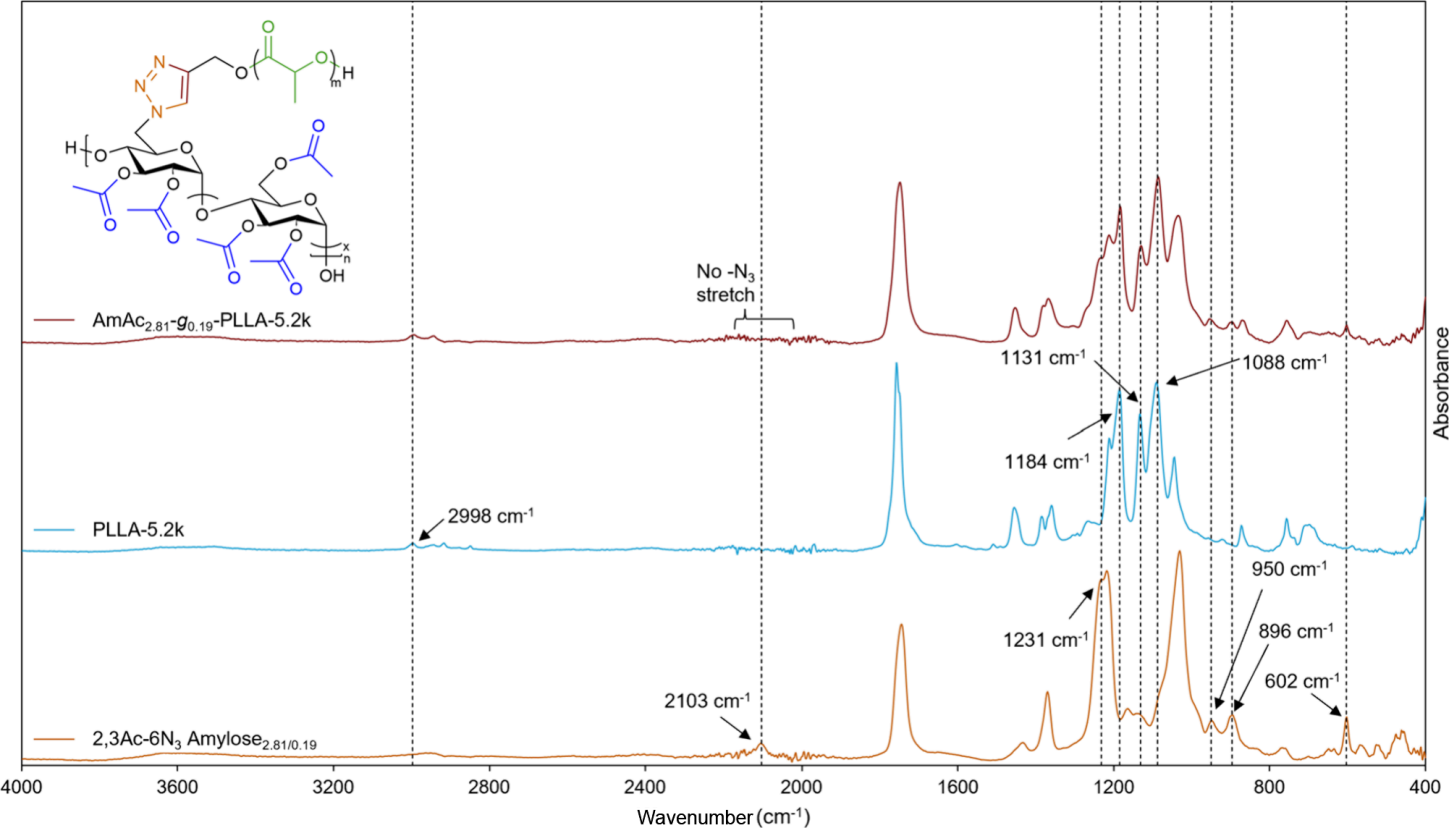
Stacked FTIR spectra of 2,3Ac-6N_3_ amylose_2.81/0.19_, PLLA-5.2k, and AmAc_2.81_-*g*_0.19_-PLLA-5.2k.

Although spectroscopic methods fully supported successful synthesis of AmAc-*g*-PLA, we sought to confirm product purity, realizing that complete separation of unreacted alkyne-terminated PLA could be challenging even after treatment with a scavenging resin. Normally, incorporation of side chains by grafting-to synthesis can be confirmed by a decrease in retention time observed in SEC,^40^ indicating an increase in hydrodynamic radius (*r*_H_) and concomitant increase in MW. However, some 2,3Ac-6N_3_ amyloses used as functionalized backbones have MW values exceeding the total exclusion range of our SEC columns.^29^ Although an increase in MW should be detected by MALS, this determination of MW assumes no aggregation of graft polymer in solution. As an alternative, we characterized our graft polymers through diffusion ordered spectroscopy (DOSY).

DOSY tracks the random diffusive motion of species in solution by monitoring the detected NMR signal as a function of gradient magnetic field strength to determine the diffusion coefficient (*D*) of a species in solution. DOSY has previously been employed to confirm synthesis of polymers with various topologies including block copolymers,^43^ multireducing-end polysaccharides,^44^ and polysaccharide graft polymers.^45^ Successful grafting-to CuAAC coupling would result in the appearance of a *D* slower than each individual graft polymer component (indicating an increase in *r*_H_), and each chemically distinct component would diffuse at the same rate (indicating covalent connection).^46^ As seen in Figure 4A, there are two distinct *D* values observed corresponding to PDLLA-7.3k (1.0·10^–10^ m^2^/s) and 2,3Ac-6N_3_ amylose_2.88/0.12_ (4.9·10^–12^ m^2^/s) when the two individual components are mixed in a common solvent. In Figure 4B, after CuAAC of 2,3Ac-6N_3_ amylose_2.88/0.12_ and PDLLA-7.3k, there is a third, distinct *D* corresponding to AmAc_2.88_-*g*_0.12_-PDLLA-7.3k (2.5·10^–12^ m^2^/s) which is slower than either individual ungrafted backbone or side chain component. At DS­(PDLLA-7.3k) 0.12, there is approximately one 7.3 kg/mol PDLLA graft every 8 repeating units, which is approximately 2.4 kg/mol of AmAc repeating units, resulting in AmAc comprising approximately 24.6% of the mass of the final graft polymer. This mass ratio means that the total molar mass of AmAc_2.88_-*g*_0.12_-PDLLA-7.3k is approximately four times the molar mass of 2,3Ac-6N_3_ amylose_2.88/0.12_. Given that *D* for AmAc_2.88_-*g*_0.12_-PDLLA-7.3k (2.5·10^–12^ m^2^/s) is approximately half the value of *D* for the 2,3Ac-6N_3_ amylose_2.88/0.12_ (4.9·10^–12^ m^2^/s), and the molar mass of AmAc_2.88_-*g*_0.12_-PDLLA-7.3k is approximately four times that of 2,3Ac-6N_3_ amylose_2.88/0.12_, *D* scales approximately to the square root of molar mass of the diffusing species. Since *D* is inversely proportional to *r*_H_ according to the Stokes–Einstein equation, the decrease in *D* after grafting-to indicates an increase in *r*_H_. This may suggest that the AmAc backbone adopts a more extended solution conformation, potentially attributable to steric and excluded volume effects from PDLLA side chains. A similar backbone stiffening effect was also observed for cellulose grafted with PS^22,24^ and PEG^24^ side chains.

**Figure 4:**
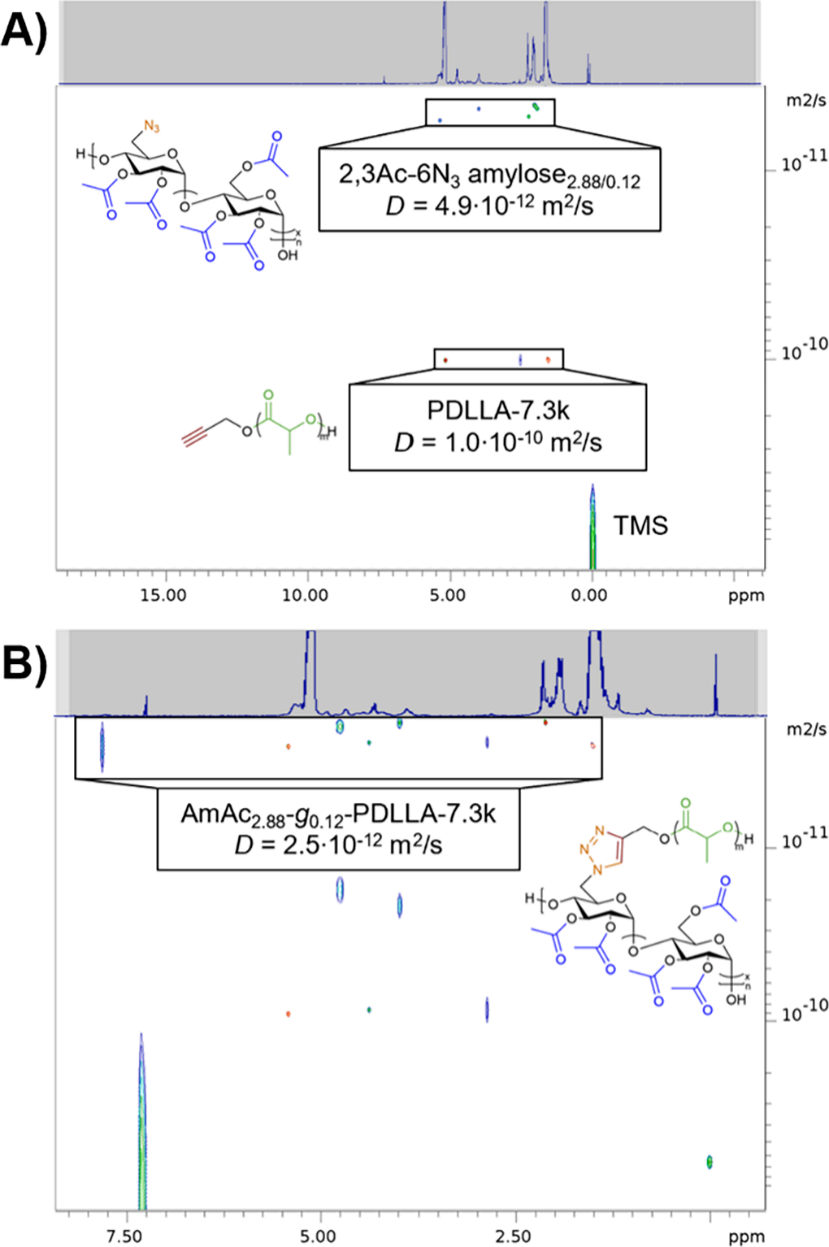
A) DOSY plot of mixture of 2,3Ac-6N_3_ amylose_2.88/0.12_ and PDLLA-7.3k, and B) DOSY plot of AmAc_2.88_-*g*_0.12_-PDLLA-7.3k.

The PLA repeating unit methyl, AmAc acetyl methyl, AmAc AGU backbone, PLA repeating unit methine, and triazole methine diffuse at the same rate, indicating successful coupling. However, there are still signals corresponding to 2,3Ac-6N_3_ amylose_2.88/0.12_ and PDLLA-7.3k, indicating that CuAAC may not have reached full conversion and not all PDLLA-7.3k was successfully removed by the scavenging resin, even though our previous spectroscopic results indicate full grafting-to conversion and PLA removal. This may have resulted from partial oxidation of the Cu­(I) catalyst to Cu­(II), which is not catalytic for CuAAC.^47^ Additionally, since we observed partial initiation of ROP of lactide by trace water, there was some nonalkyne functionalized PLA introduced into the reaction mixture for CuAAC. This lack of chain-end functionality can explain the incomplete removal of PLA, as this nonfunctionalized PLA is unreactive to CuAAC for both grafting-to and resin scavenging, Although this result was not intended, the presence of the distinct *D* for AmAc_2.88_-*g*_0.12_-PDLLA-7.3k indicates successful grafting-to CuAAC.

### 3.4 Effect of Composition on Thermal Properties of AmAc-*g*-PLLA and AmAc-*g*-PDLLA

The impact of covalent conjugation of PLA to AmAc on thermal properties was investigated using DSC. The *T*_g_ of alkyne-terminated PDLLA ranged from 50–55 °C and the *T*_g_ of alkyne-terminated PLLA ranged from 48–56 °C, with alkyne-terminated PLLA exhibiting melting endotherms from 142–151 °C. DSC thermograms of semicrystalline PLLA samples exhibited a bimodal melting endotherm commonly observed for PLLA that can be attributed to melt-recrystallization.^48,49^ Our results for the relationship between *M*_n_ and *T*_g_ of both PLA isomers follow a Fox–Flory relationship, with extrapolated values of *T*_g,∞_ of 55 and 57 °C for PDLLA and PLLA, respectively, in agreement with previous literature results (Figures S6 and S7, Table S1).^50,51^ Although PDLLA and PLLA are isomeric, the higher *T*_g,∞_ of PLLA can be rationalized by considering the effects of crystallinity on available free volume and chain mobility. Since the Fox–Flory relationship suggests that chain ends contribute significantly more to available free volume than interior repeat units, *T*_g_ increases with increasing *M*_n_, as there will be fewer end groups relative to repeat units. Coupled with the fact that PLLA is semicrystalline while PDLLA is amorphous, it can be rationalized that some PLLA crystallites will act as physical cross-links and limit chain mobility of the amorphous phase, increasing *T*_g_ (since the glass transition is a phenomenon associated only with the amorphous phase).

Grafting-to 2,3Ac-6N_3_ amylose by CuAAC significantly impacted the thermal behavior of PLA side chains in AmAc-*g*-PLA graft polymers. As seen in Figure 5A, the *T*_g_ of PLLA side chains in AmAc_2.88_-*g*_0.12_-PLLA-10.6k decreased from 52 to 48 °C while the *T*_m_ decreased from 148 to 136 °C for ungrafted and grafted side chains, respectively. PLLA grafts of AmAc_2.96_-*g*_0.04_-PLLA-18.9k also exhibited a reduction of *T*_g_ and *T*_m_ compared to ungrafted counterparts (Figure S8). A similar depression of side chain *T*_m_ compared to ungrafted homopolymer was reported for CTA-*g*-PLLA^12^ and ethyl cellulose-*g*-poly­(*p*-dioxanone).^52^ This melting point depression can be rationalized by the presence of the rigid AmAc branching points and increased local concentration of PLLA end groups, both of which may act as defects and prevent formation of larger crystallites that melt at higher temperatures. Similar results were also observed for PLLA star polymers, where both *T*_g_ and *T*_m_ were suppressed compared to linear PLLA.^53^

**Figure 5:**
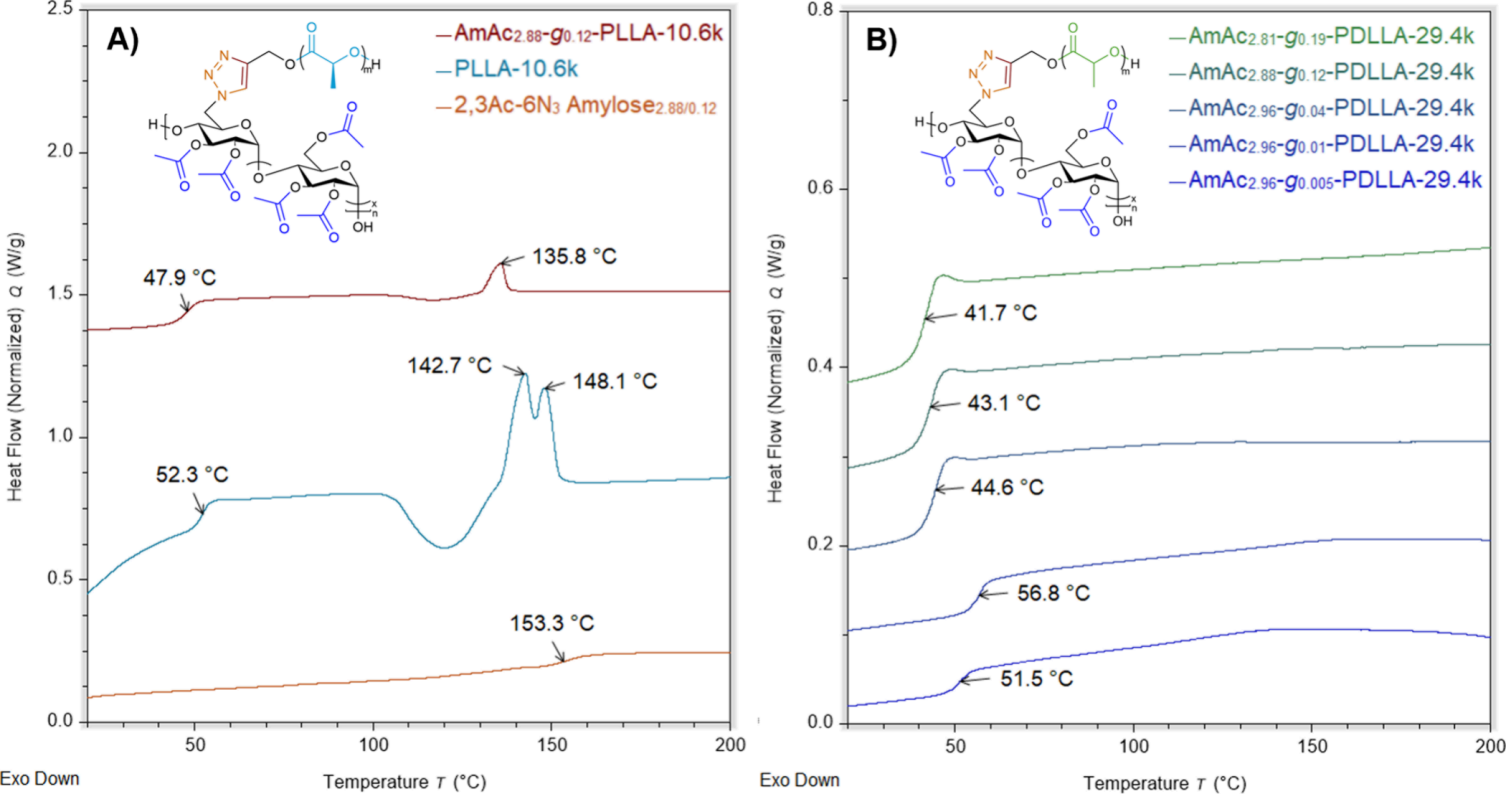
A) Stacked DSC thermograms of 2,3Ac-6N_3_ amylose_2.88/0.12_, PLLA-10.6k, and AmAc_2.88_-*g*_0.12_-PLLA-10.6k, and B) Stacked DSC thermograms of AmAc-*g*-PDLLA-29.4k with varying graft density.

No PLLA melting endotherms were observed for AmAc_2.81_-*g*_0.19_-PLLA-5.2k or AmAc_2.88_-*g*_0.12_-PLLA-8.5k, suggesting that there is a critical combination of PLLA length and graft density required for grafted side chains to maintain crystallizability (Figures S9 and S10). This observation is consistent with previous studies of cellulose esters grafted with polyhydroxyalkanoates.^54^ This could suggest that tethering crystallizable PLLA segments to the rigid AmAc backbone could impair PLLA molecular mobility enough to prevent generation of the regular packing structure necessary for crystallization. In the case where PLLA grafts did not exhibit melting endotherms, the *T*_g_ of PLLA segments increased from 49 to 58 °C and 52 to 63 °C for AmAc_2.81_-*g*_0.19_-PLLA-5.2k or AmAc_2.88_-*g*_0.12_-PLLA-8.5k, respectively, both of which exceed the calculated *T*_g,∞_ predicted by the Fox–Flory relationship. A similar PLLA graft crystallization inhibition and increase of PLLA *T*_g_ was observed for xylan-*g*-PLLA graft polymers.^34^ Since one PLLA end group is tethered to the AmAc backbone, and polymer end groups contribute more to free volume than interior repeating units, the reduction in free volume afforded by a PLLA end group could cause *T*_g_ to increase.

As seen in Figure 5B, *T*_g_ of PDLLA side chains of AmAc-*g*-PDLLA-29.4k graft polymers increased with decreasing graft density, with AmAc_2.81_-*g*_0.19_-PDLLA-29.4k exhibiting a PDLLA *T*_g_ at 42 °C and AmAc_2.96_-*g*_0.005_-PDLLA-29.4k exhibiting a PDLLA *T*_g_ at 52 °C, with the highest PDLLA *T*_g_ of 57 °C for AmAc_2.96_-*g*_0.01_-PDLLA-29.4k. PBD-*g*-PLLA^55^ and poly­(β-myrcene)-*g*-PLLA^56^ exhibited similar trends of decreasing PLA *T*_g_ with increasing graft density. While this may seem contradictory to previous results, there are multiple structural variables at play that dictate the molecular mobility of grafted side chains. Two competing factors contribute to the glass transition of polymer side chains in graft polymers: (1) the increase in free volume afforded by chain ends of polymer grafts, favoring *T*_g_ suppression, and (2) the restriction of segmental mobility arising from covalent attachment to a polymer backbone, favoring *T*_g_ enhancement. Additionally, the flexibility of the graft polymer backbone is affected by topological factors such as graft length and graft density,^57^ which may in turn also change the *T*_g_ of grafted side chains. Furthermore, the total weight fraction of PLA side chains compared to AmAc backbone will change with varying graft length and graft density, which may also affect the thermal properties of the graft polymer containing PLA arms connected to an AmAc backbone that are mutually immiscible (Table S2). These complex interrelationships between graft polymer topology and thermal properties may warrant further investigation and could provide valuable guidance for designing well-defined polysaccharide graft polymers.

Surprisingly, there was no distinct *T*_g_ associated with the AmAc backbone of any AmAc-*g*-PLA graft polymers investigated. The lower weight fraction of the AmAc backbone (5.1–27.7 wt %) relative to PLA side chains may inhibit AmAc association and reduce the intensity of the glass transition as measured by DSC. Even for graft polymers with higher AmAc weight fractions (49.5–66.2 wt %) the glass transition was difficult to observe, potentially due to already broad glass transition exhibited by 2,3Ac-6N_3_ amylose compared to that of PLA. Previous polysaccharide graft polymers also displayed difficult to detect polysaccharide backbone glass transitions.^12,34^ Thermal stability of AmAc-*g*-PDLLA graft polymers, as measured by TGA, depended on PDLLA grafting density. Pure PDLLA and StAc exhibited the onset of thermal degradation at 277 and 337 °C, respectively. The thermal stability of AmAc-*g*-PDLLA-29.4k increased with decreasing grafting density, with AmAc_2.81_-*g*_0.19_-PDLLA-29.4k and AmAc_2.96_-*g*_0.005_-PDLLA-29.4k exhibiting thermal degradation beginning at 266 and 324 °C, respectively (Figure S11). This increase in thermal stability may be useful when preparing blends through extrusion, allowing increased operating temperature to permit manageable melt viscosities of the StAc blend component.

### 3.5 Initial Investigation into AmAc-*g*-PDLLA as Compatibilizer for StAc/PDLLA Blends

Having successfully synthesized AmAc-*g*-PLA and determined the impact of polymer grafting on its thermal properties, we initiated investigations of the ability of AmAc-*g*-PLA to compatibilize immiscible blends of StAc and PLA. As both StAc^58^ and PLA^59^ are biodegradable (under appropriate composting conditions) and are sourced from renewable starch feedstocks, blending them could create appealing sustainable materials. PLA possesses favorable physical properties similar to traditional commodity plastics such as PS and poly­(ethylene terephthalate) (*T*_g_ = 45–65 °C, *T*_m_ = 150–200 °C, σ = 110 MPa, E = 3.3 GPa), but is generally hindered by its higher production cost relative to petroleum-based plastics.^60^ On the other hand, StAc is inexpensive but compromised by brittleness arising largely from its amylopectin content.^61^ As such, a compatibilized StAc/PLA blend could be a low-cost alternative to PLA, particularly for applications in single-use packaging or biodegradable cutlery.

While compatibilized blends of PLA and TPS (i.e., starch containing a plasticizer such as glycerol or water) have been extensively studied,^62^ there is less precedent for studying StAc/PLA blends.^63^ Various factors affect the miscibility of StAc and PLA, including DS­(Ac), MW of both polymers, stereochemistry of PLA, and potentially the amylose vs amylopectin content of the StAc. For our initial proof-of-concept study to investigate the ability of AmAc-*g*-PLA as a compatibilizer, we decided to simplify our test system. We limited our starch derivative to a high DS­(Ac) StAc, as this generated a polysaccharide derivative soluble in CHCl_3,_ which is a good solvent for PLA and volatile enough for facile film casting. Film casting blends was more appealing for proof-of-concept studies than extrusion, as significantly less sample was required for analysis, although many starch/PLA and StAc/PLA blends previously studied were prepared through extrusion.^18,39,62–65^ It should also be noted that starch or starch ester blends with PLA prepared by extrusion may be further complicated by partial thermal esterification of available starch OH groups by PLA, which is often not acknowledged as a possibility and/or not quantified. We treated starch with AcOH and TFAA to generate StAc with DS­(Ac) 2.94 and high MW (*M*_n_ = 364 kg/mol, *Đ* = 4.09). This method was adapted from an existing procedure and relies upon generation of mixed acetic/trifluoroacetic acid anhydride *in situ* as the active acylating agent.^66^ While studying blends of both StAc/PLLA and StAc/PDLLA will be valuable, we chose to limit our initial investigation to amorphous StAc/PDLLA blends to eliminate the effects of crystallization on compatibilization. We synthesized PDLLA (*M*_n_ = 93.9 kg/mol, *Đ* = 1.63) of comparable *M*_n_ and *Đ* to that of commercially available PLLA from Natureworks (*M*_n_ = 85.7 kg/mol, *Đ* = 1.69, as determined from SEC in THF) through bulk ROP of D,l-lactide using BnOH as initiator and SnOct_2_ as catalyst (Scheme 2).

**Scheme 2:**
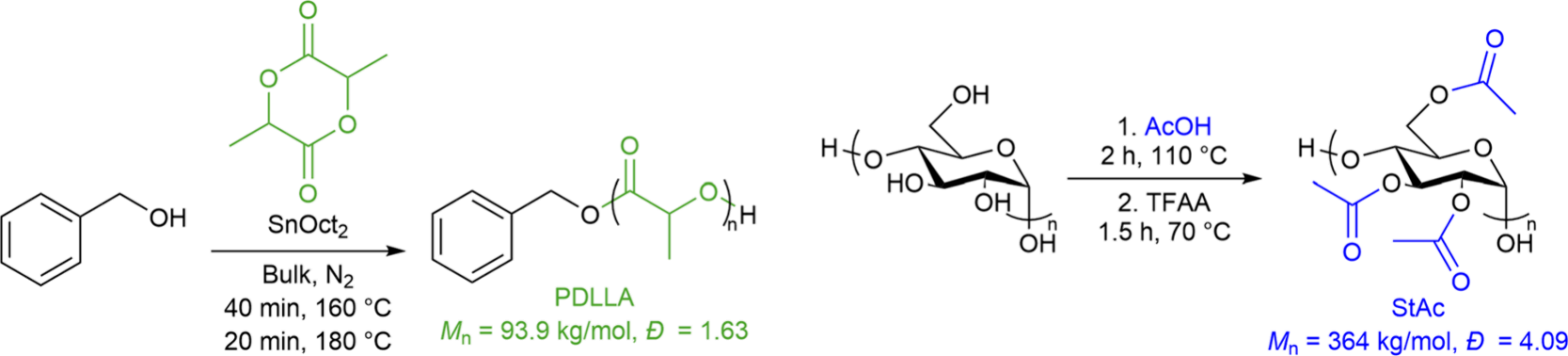
Synthesis of StAc and PDLLA Used for Solution-Cast Blend Compatibilization Studies

We separately dissolved the desired amounts of StAc and PDLLA in CHCl_3_, mixed the solutions, then drop-cast films on glass slides and let the solvent slowly evaporate, ensuring formation of a smooth film. We chose to study blends with a composition of 70/30 StAc/PDLLA, as this generated blends with average interdomain spacing (as determined by SALLS) of 4.4 ± 0.5 μm. Looking at the PCOM image in Figure 6A, the phase-separated morphology of the base blend exhibits a texture characteristic of spinodal decomposition, indicating thermodynamic immiscibility between StAc and PDLLA. In general, strong compatibilization is observed when interdomain spacings are below 1.0 μm, so the presence of uncompatibilized blends with domain sizes near 5.0 μm gave us a wide size range to observe the effects of AmAc-*g*-PDLLA on interdomain spacing using SALLS and PCOM.

**Figure 6:**
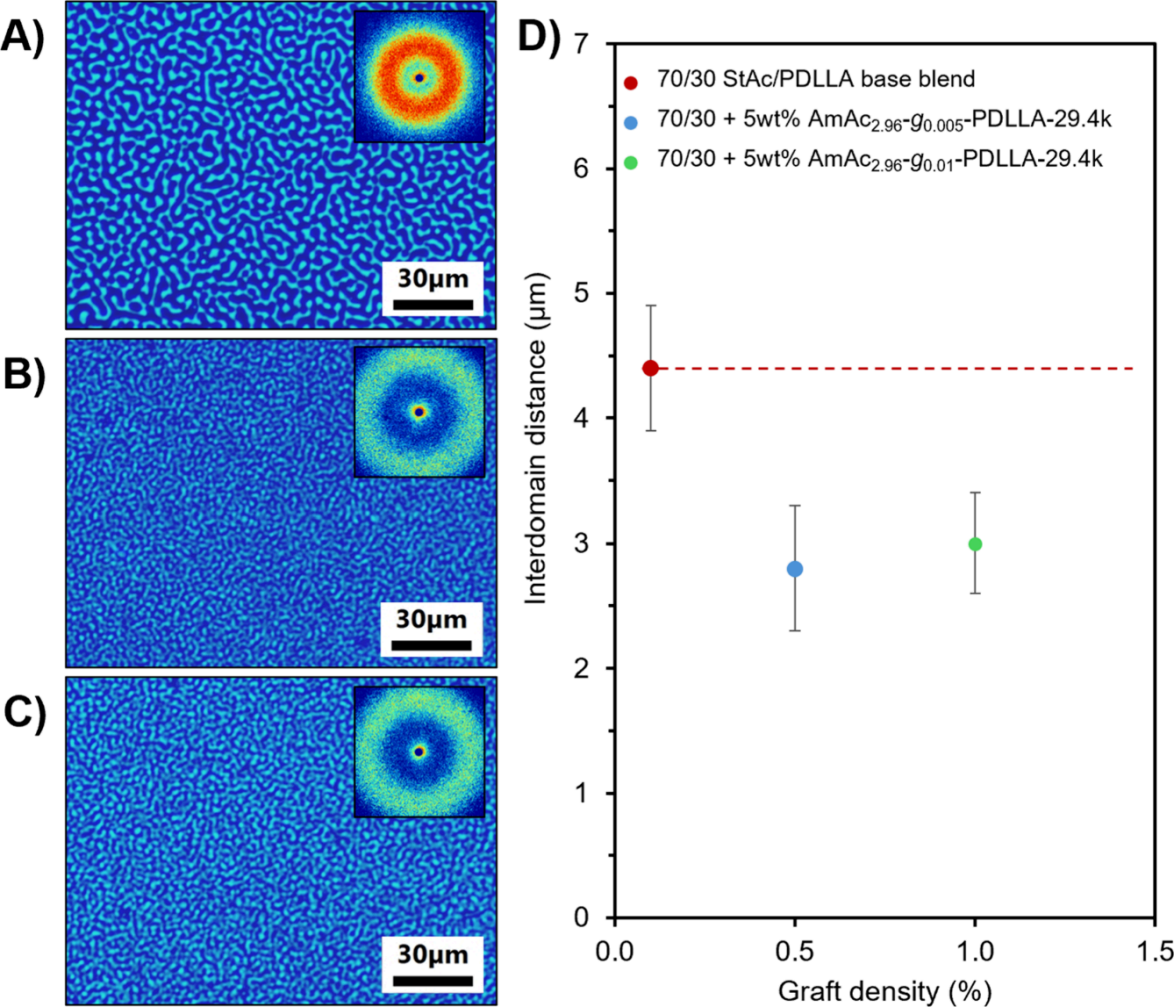
PCOM images and SALLS scattering patterns of 70/30 StAc/PDLLA blends with A) no graft copolymer, B) 5 wt % AmAc_2.96_-*g*_0.005_-PDLLA-29.4k, and C) 5 wt % AmAc_2.96_-g_0.01_-PDLLA-29.4k, and D) plot of interdomain distance of StAc/PDLLA blends obtained from SALLS as a function of AmAc-*g*-PDLLA graft density.

For our initial investigation into the ability of AmAc-*g*-PDLLA graft polymers to compatibilize immiscible blends, we prepared blends of 70/30 StAc/PDLLA containing 5 wt % of low graft density AmAc-*g*-PDLLA graft polymers containing PDLLA-29.4k side chains. As seen in Figures 6B–D, both AmAc_2.96_-*g*_0.005_-PDLLA-29.4k and AmAc_2.96_-*g*_0.01_-PDLLA-29.4k graft polymers (prepared through grafting-to CuAAC using 0.125 and 0.25 eq PDLLA per 2,3Ac-6N_3_ amylose_2.96/0.04_, respectively) reduced interdomain spacing to 2.8 ± 0.5 μm and 3.0 ± 0.4 μm, respectively. This reduction of domain size indicates that AmAc-*g*-PDLLA can reduce interfacial tension between immiscible StAc and PDLLA phases and stabilize the phase-separated morphology of StAc/PDLLA blends. With a library of AmAc-*g*-PDLLA graft polymers with varying graft lengths and graft densities in hand, we envision the ability to sensitively tune compatibilizer performance by understanding the effects of graft polymer topology. We are pursuing this promising direction in an ongoing study by systematically varying graft polymer structure to elucidate the effect of compatibilizer topology on the morphology of immiscible blends. We believe that deepening the understanding of compatibilizer performance in biobased blends will permit generation of valuable materials for commercial applications, such as compostable cutlery and degradable packaging.

## 4 Conclusions

We have demonstrated the ability to generate well-defined AmAc-*g*-PLA graft polymers via grafting-to CuAAC with control over grafting position, length, density, and stereochemistry. *T*_g_ values of PLA side chains tended to increase after conjugation to the AmAc backbone, while AmAc-*g*-PLLA exhibited thermal transitions indicating a semicrystalline nature of PLLA grafts. AmAc-*g*-PDLLA graft polymers with grafting densities ranging from 0.5% to 19% and graft lengths ranging from 5.2–29.4 kg/mol were prepared as potential compatibilizers for immiscible StAc/PDLLA blends. Interdomain spacing in StAc/PDLLA blends, as observed through SALLS and PCOM, was decreased with the addition of 5 wt % of AmAc-*g*-PDLLA, indicating the potential value of these graft polymers as blend compatibilizers. An in-depth structure–property relationship study between AmAc-*g*-PDLLA topology and StAc/PDLLA blend morphology will soon be reported.

The new graft polymers and the blends that they have enabled will have considerable value as biomaterials. They are highly likely to be biodegradable in composting environments, being based entirely on components like PLA and StAc that are known themselves to biodegrade in such environments. These biodegradable materials are expected to have better performance than immiscible blend-based starch/PLA materials now in use in applications like cutlery and small shampoo bottles, thereby taking their place as much more widely applicable, biofriendly plastics and packaging materials, especially for single use applications. Our further studies on structural factors that impact performance of these graft polymers as PLA/StAc compatibilizers will be reported in depth in an upcoming publication. As these blends and compatibilizers comprise materials that also have been applied *in vivo*, they may also have promise in biomedical applications.

## Supplementary Material



## Notes

Degree of substitution (DS) calculation equations; Stejskal-Tanner and Fox–Flory equations; equation for calculating interdomain distance by small-angle laser light scattering (SALLS); procedure for preparation of azide scavenging resin; FTIR, 1H NMR, and 13C NMR spectra of AmAc-*g*-PLA graft polymers; representative DSC thermograms of synthesized PDLLA; plot of Tg vs Mn and Tg vs 1/Mn for synthesized PLLA and PDLLA; table of Fox–Flory parameters for PLLA and PDLLA; representative DSC thermograms of PLLA, 2,3Ac-6N3 amylose, and AmAc-*g*-PLLA, table of thermal properties and compositions of synthesized AmAc-*g*-PLA samples; and TGA thermograms for blend components and synthesized AmAc-*g*-PDLLA samples (PDF)
