# Supplementary material for: Well-Defined Amylose Acetate-graft-polylactide Graft Polymers as Compatibilizers for Renewable Polymer Blends
Source: Biomacromolecules. 2025 Sep 26;26(10):6893–905. doi: 10.1021/acs.biomac.5c01188 (PMC12522131; doi:10.1021/acs.biomac.5c01188)
Supplement: Supplementary file 1 [file bm5c01188_si_001.pdf]

Supplementary Information:

Well-Defined Amylose Acetate-*graft*-Polylactide Graft Polymers as Compatibilizers for Renewable Polymer Blends

Jeffrey E. Thompson,[1] Isabela T. Coutinho,[1] Nicholas F. Pietra,[1] Louis A. Madsen,[1,2] Robert B. Moore,[1,2] & Kevin J. Edgar[1,3]*

1. *Macromolecules Innovation Institute, Virginia Tech, Blacksburg, VA 24061, United States*
2. *Department of Chemistry, Virginia Tech, Blacksburg, VA 24061, United States*
3. *Department of Sustainable Biomaterials, Virginia Tech, Blacksburg, VA 24061, United States*

*Corresponding author: Kevin J. Edgar – Macromolecules Innovation Institute and Department of Sustainable Biomaterials, Virginia Tech, Blacksburg, VA 24061, United States, Email: kjedgar@vt.edu

Number of pages: 9

Number of equations: 4

Number of figures: 11

Number of tables: 2

Number of procedures: 1

Calculations

$$1)\quad DS(PLA) = \frac{I(triazole - CH)}{I(AmAc\ H3)}$$

The degree of substitution of PLA (DS(PLA)) grafted to AmAc was calculated using Equation 1, where I(triazole -CH) is the integral value of the triazole formed after CuAAC (7.79 ppm) and

I(AmAc H3) is the integral value of H3 of the AmAc AGU (5.37 ppm), obtained from ^{1}H NMR in $CDCl_3$.

$$2)\quad I = I_o exp\left(-D\gamma^2 g^2 \delta^2 \Delta - \frac{\delta}{3}\right)$$

Self-diffusion coefficients of selected polymers were obtained using the pulse-gradient stimulated echo (PGSTE) experiment on ^{1}H nuclei at 25 °C. The Stejskal-Tanner equation (Equation 2) was used to fit the measured signal amplitude (I) as a function of gradient strength (g), where I_o is the signal amplitude at $g = 0$, γ is the gyromagnetic ratio of the measured nucleus, δ is the effective gradient pulse duration, Δ is the diffusion times between gradient pulses, and D is the self-diffusion coefficient.[1]

$$3)\quad T_g = T_{g,\infty} - \frac{K}{M_n}$$

The relationship between number-average molecular weight (M_n) and the glass transition temperature (T_g) can be described by the Fox-Flory equation (Equation 3), where $T_{g,\infty}$ is the glass transition temperature at infinite molecular weight and K is a constant.[2]

$$4)\quad d = \frac{2\pi}{q}$$

The interdomain distance (d) of immiscible StAc/PDLLA blends was calculated by according to Equation 4, where q is the scattering vector and d is the interdomain distance at the maximum intensity.

Preparation of azide-functionalized polystyrene resin (PS-N_3)

To an oven-dried 250 mL flask equipped with a magnetic stir bar, Merrifield resin (2.50 g, ~6.25 mmol -Cl/g) was suspended in 125 mL anhydrous DMSO. NaI (4.06 g, 62.5 mmol, 10 eq) and NaN_3 (2.81 g, 18.8 mmol, 3 eq) were added to the flask and dissolved. The mixture was heated to 80 °C and stirred at that temperature for 48h. The flask was then allowed to cool to RT, filtered, and rinsed with 1L DI H_2O. The beads were then washed with a sequence of H_2O,

acetone, $CHCl_3$, acetone, and H_2O to swell crosslinked PS and remove any residual salts present. The beads were then collected, air-dried, and dried under reduced pressure at 50 °C overnight.

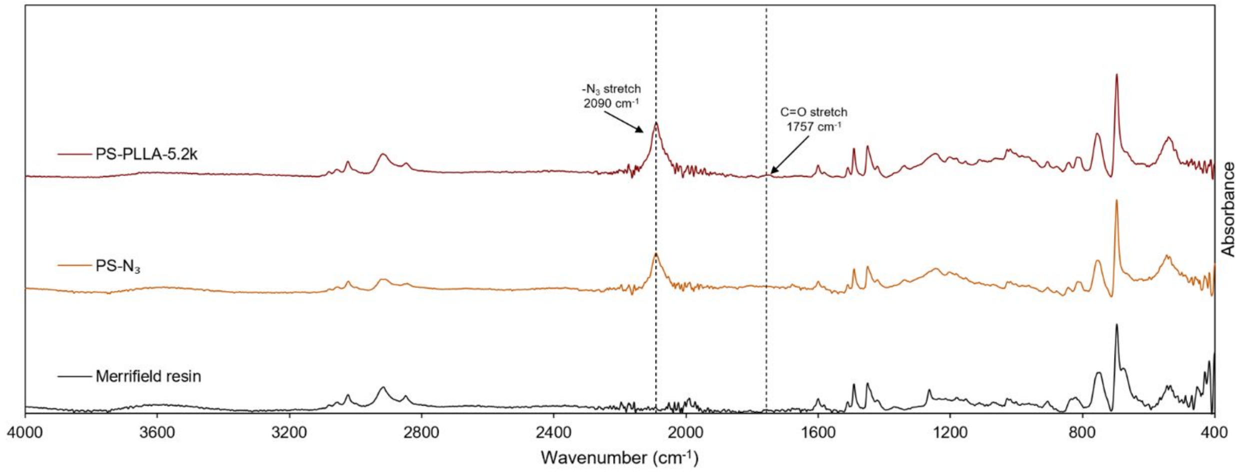

Figure S1: Stacked FTIR spectra of unmodified Merrifield resin, $PS\text{-}N_3$ resin, PLLA-10.6k, and $PS\text{-}N_3$ resin after scavenging PLLA-10.6k

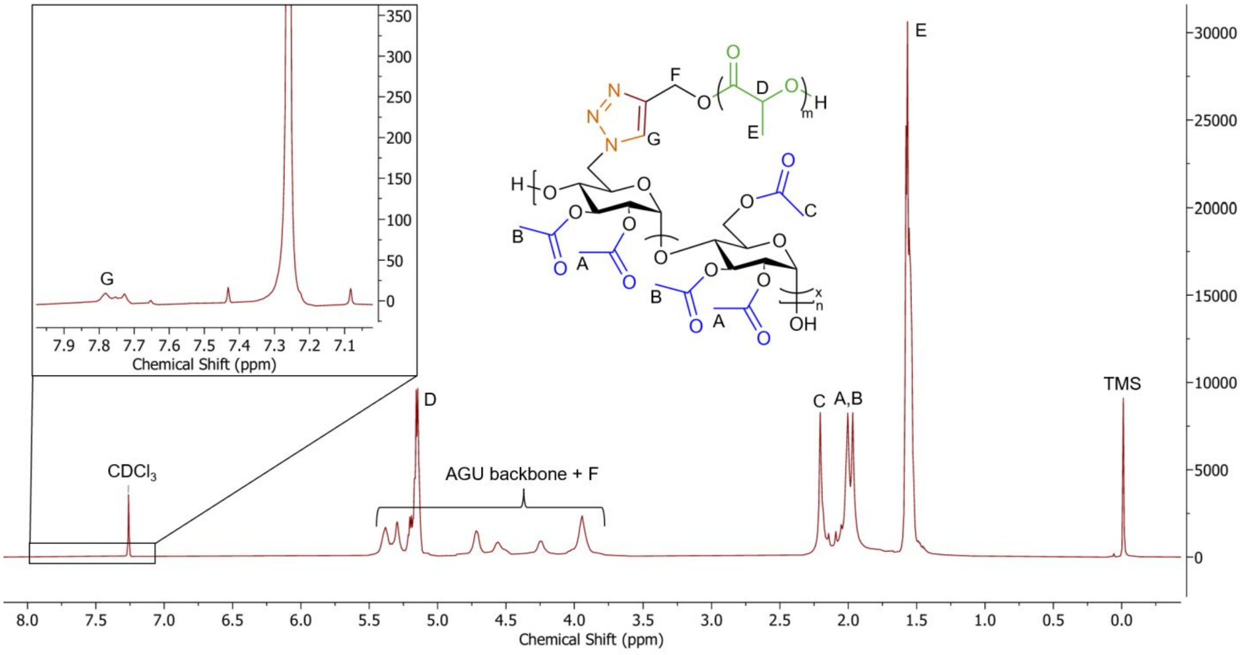

Figure S2: ^{1}H NMR spectrum of $AmAc_{2.96}$-$g_{0.01}$-PDLLA-29.4k

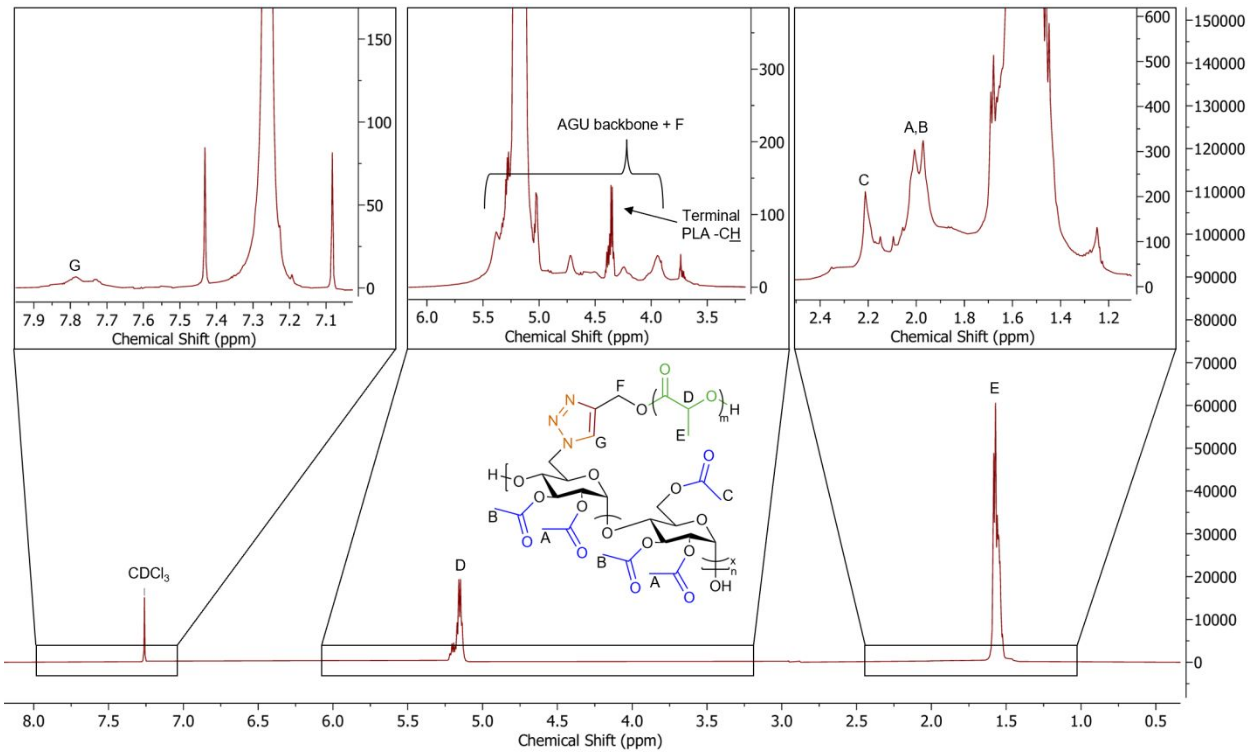

Figure S3: ^{1}H NMR spectrum of AmAc$_{2.81}$-$g_{0.19}$-PDLLA-29.4k

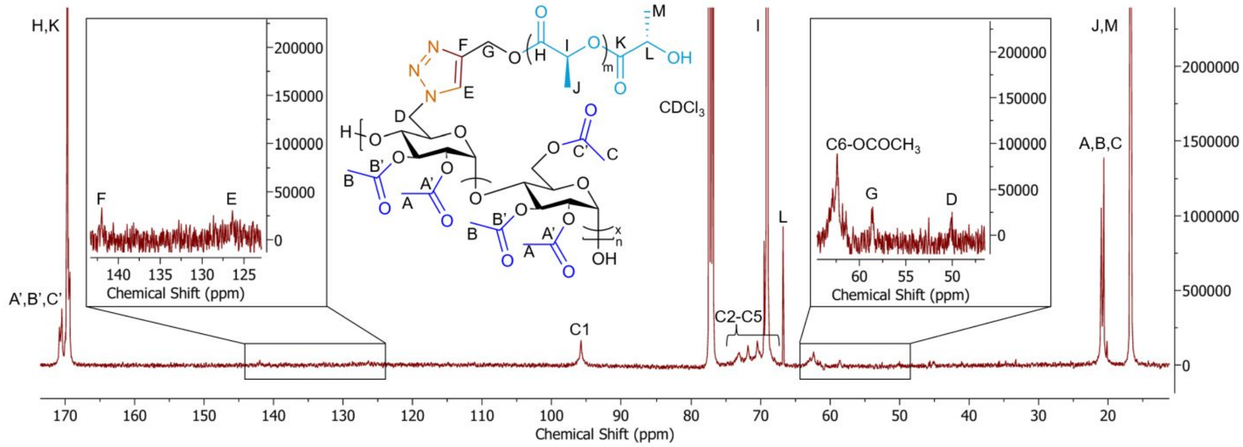

Figure S4: ^{13}C NMR spectra of AmAc$_{2.81}$-$g_{0.19}$-PLLA-5.2k

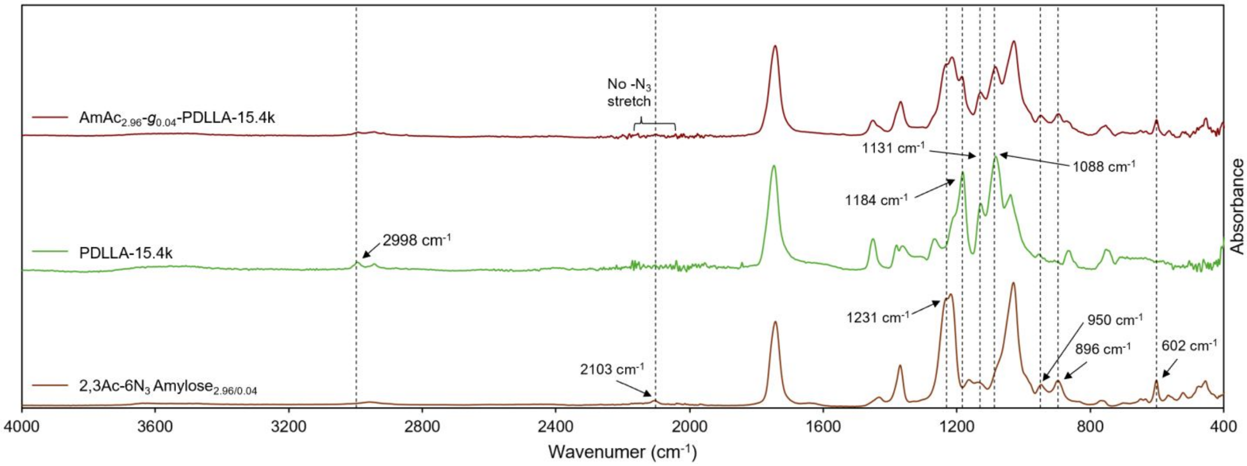

Figure S5: Stacked FTIR spectra of 2,3Ac-6N_3 amylose$_{2.96/0.04}$, PDLLA-15.4k, and AmAc$_{2.96}$-*g*$_{0.04}$-PDLLA-15.4k

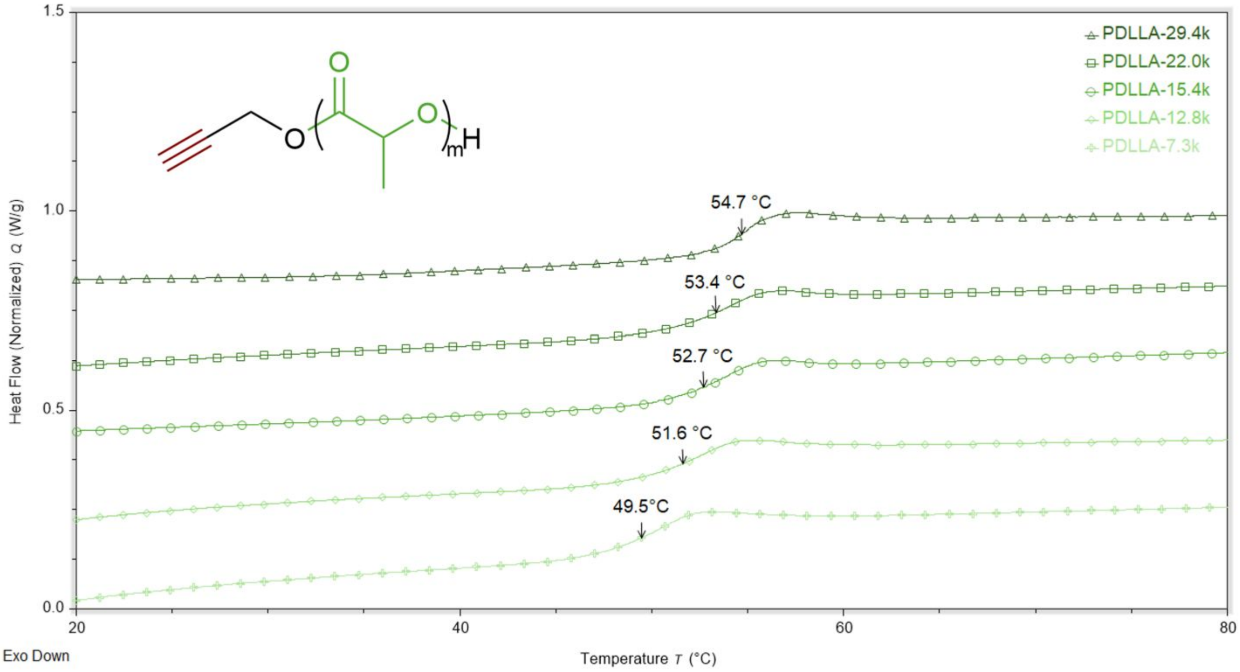

Figure S2: Stacked DSC thermograms of alkyne-terminated PDLLA with varying M_n

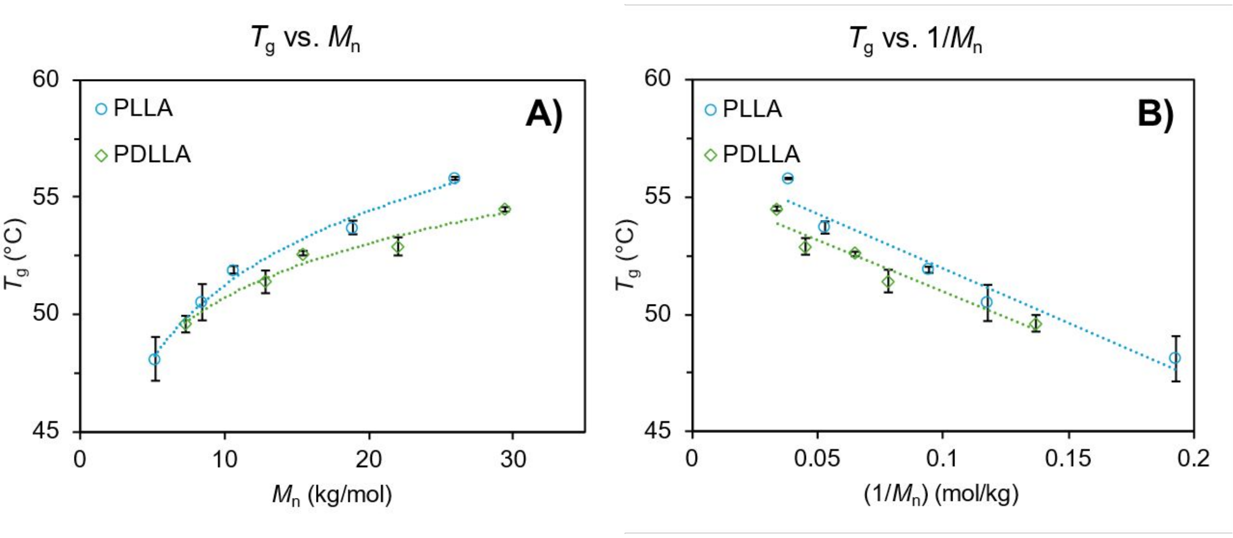

Figure S3: A) Plot of T_g vs. M_n for PLLA and PDLLA. B) Fox-Flory plot of $1/T_g$ vs. M_n for PLLA and PDLLA

Table S1: Experimentally-determined Fox-Flory parameters of PLLA and PDLLA

**Polymer**	**$T_{g,\infty}$ (°C)**	**K (kg/mol)**
PLLA	56.7	46.8
PDLLA	55.3	43.8

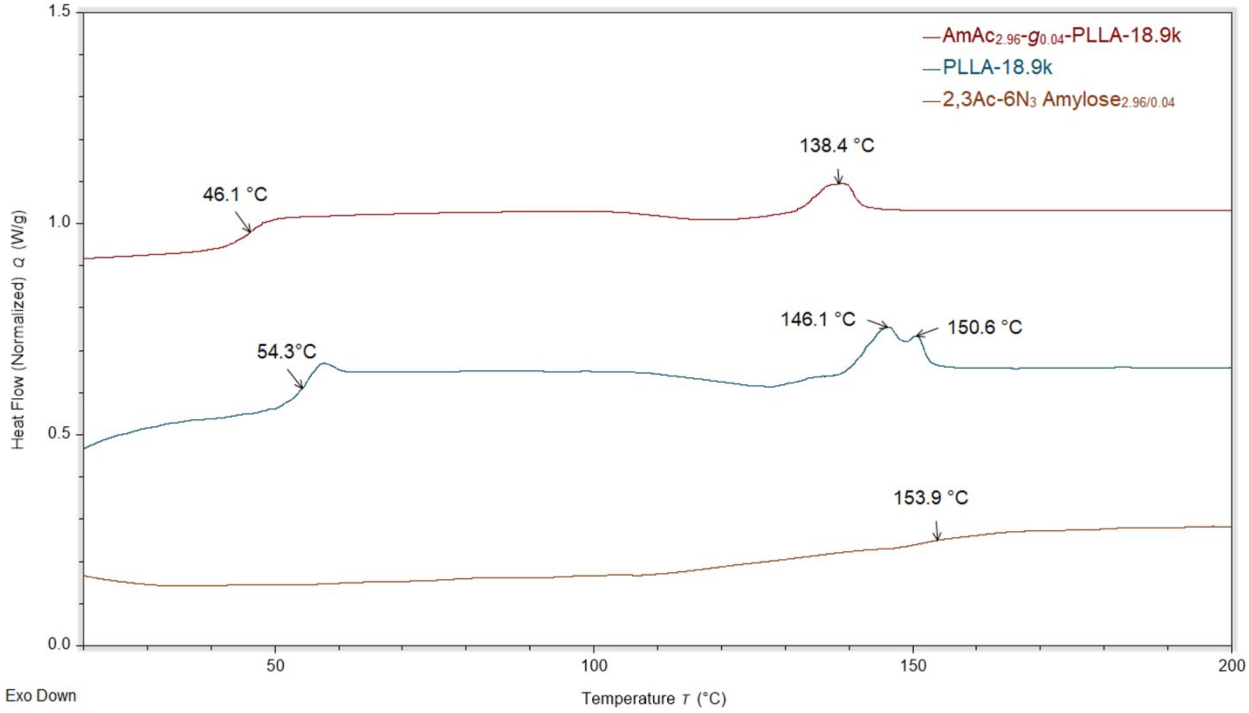

Figure S4: Stacked DSC thermograms of 2,3Ac-6N_3 amylose$_{2.96/0.04}$, PLLA-18.9k, and AmAc$_{2.96}$-$g_{0.04}$-PLLA-18.9k

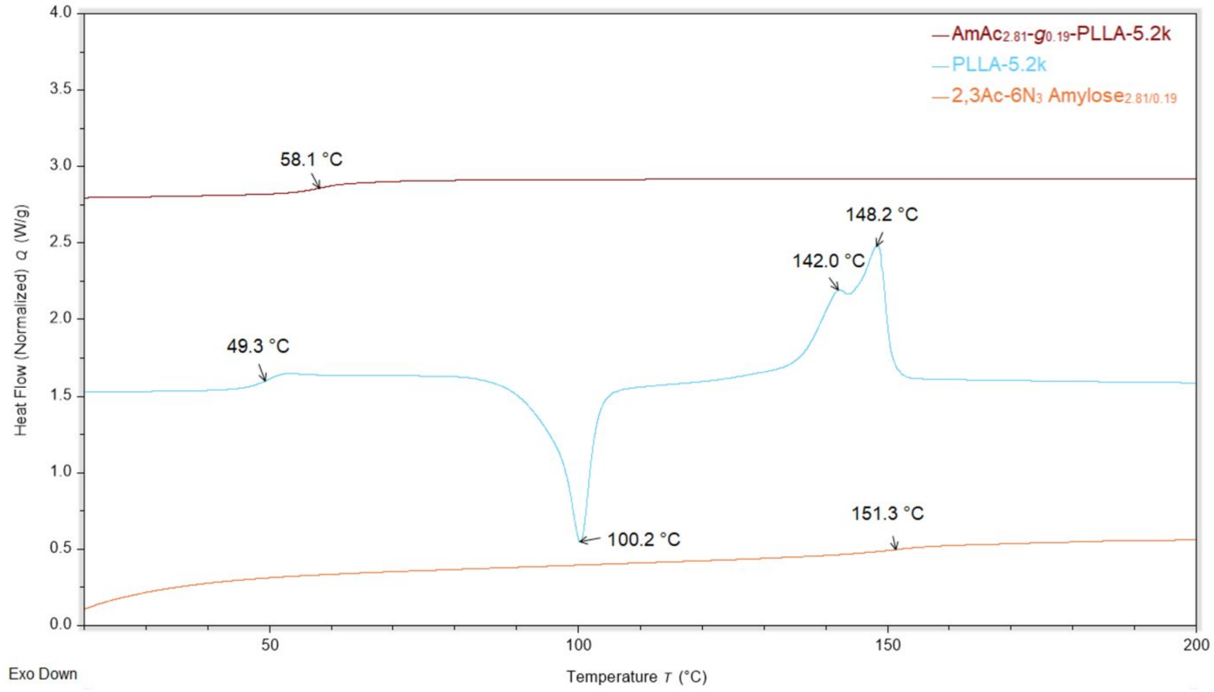

Figure S5: Stacked DSC thermograms of 2,3Ac-6N_3 amylose$_{2.81/0.19,}$ PLLA-5.2k, and AmAc$_{2.81}$-$g_{0.19}$-PLLA-5.2k

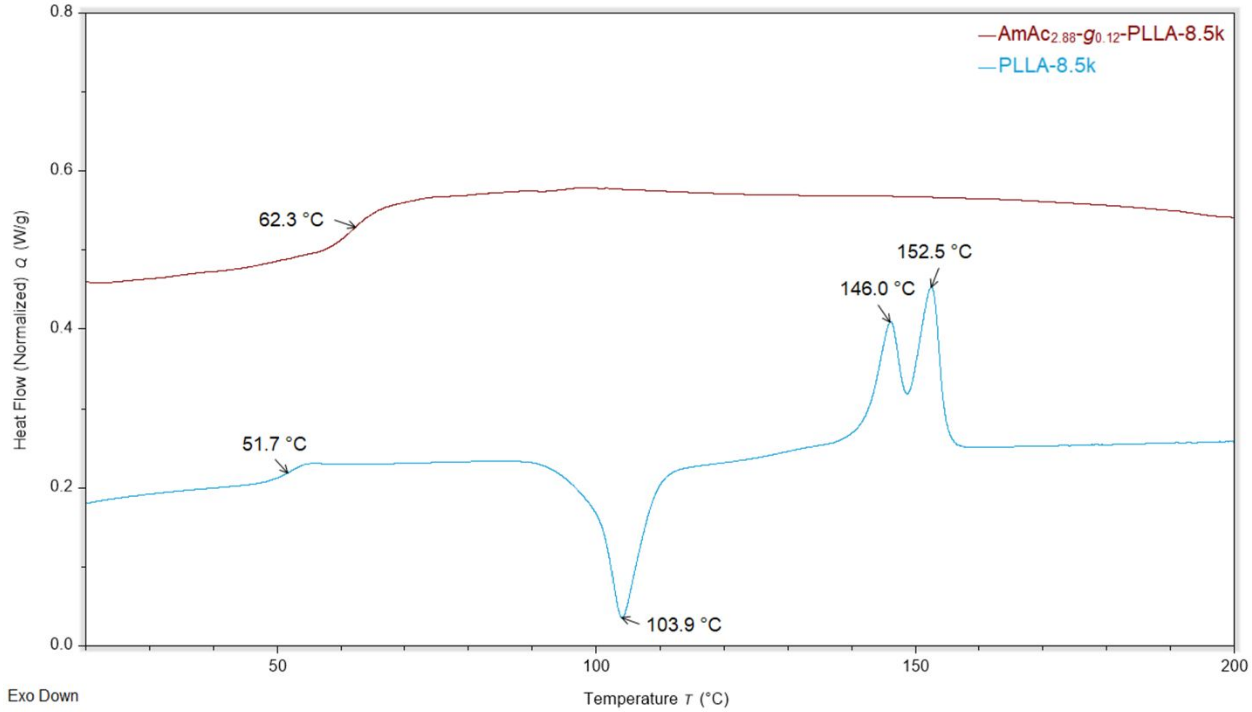

Figure S6: Stacked DSC thermograms of PLLA-8.5k and AmAc$_{2.88}$-$g_{0.12}$-PLLA-8.5k

Table S2: Thermal properties of PLA grafts after grafting-to synthesis of AmAc-g-PLA

**Sample**	**PLA $T_{g,graft}$ (°C)**	**ΔT_g (°C)**	**PLA wt%**	**AmAc wt%**
AmAc$_{2.81}$-$g_{0.19}$-PDLLA-29.4k	41.7	-12.8	94.9	5.1
AmAc$_{2.88}$-$g_{0.12}$-PDLLA-29.4k	43.1	-11.4	92.3	7.7
AmAc$_{2.88}$-$g_{0.12}$-PLLA-10.6k	47.9	-4.4	81.3	18.7
AmAc$_{2.96}$-$g_{0.04}$-PDLLA-29.4k	44.6	-9.9	80.2	19.8
AmAc$_{2.88}$-$g_{0.12}$-PLLA-8.5k	62.3	10.6	77.7	22.3
AmAc$_{2.81}$-$g_{0.19}$-PLLA-5.2k	58.1	8.8	76.9	23.1
AmAc$_{2.96}$-$g_{0.04}$-PLLA-18.9k	46.1	-8.2	72.3	27.7
AmAc$_{2.96}$-$g_{0.01}$-PDLLA-29.4k	56.8	2.3	50.5	49.5
AmAc$_{2.96}$-$g_{0.005}$-PDLLA-29.4k	51.5	-3	33.8	66.2

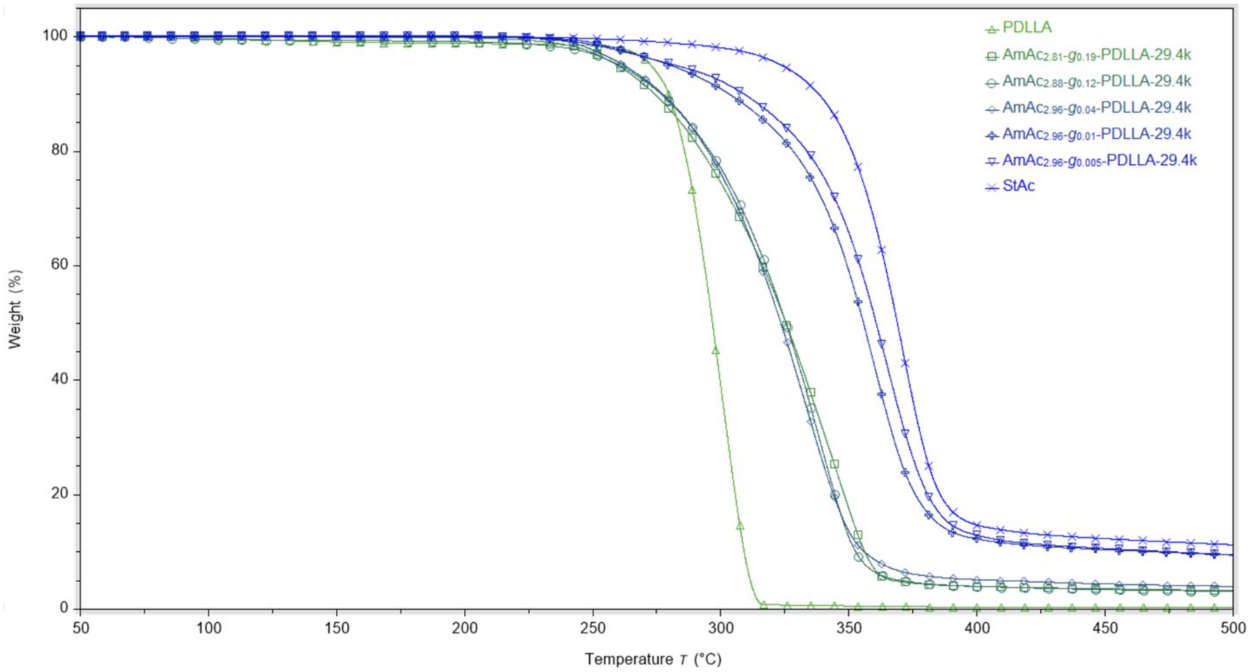

Figure S7: Stacked TGA thermograms of PDLLA, StAc, and AmAc-*g*-PDLLA-29.4k with varying graft density

References

1. Fox, T.G.; Flory, P.J. Second-Order Transition Temperatures and Related Properties of Polystyrene. I. Influence of Molecular Weight. *J. Appl. Phys.,* **1950**, *21,* 581-591.

2. Stejskal, E.O.; Tanner, J.E. Spin Diffusion Measurements: Spin Echoes in the Presence of a Time-Dependent Field Gradient. *J. Chem. Phys.,* **1965**, *42* (1), 288-292.
